# Patient-Generated Health Data (PGHD): Understanding, Requirements, Challenges, and Existing Techniques for Data Security and Privacy

**DOI:** 10.3390/jpm14030282

**Published:** 2024-03-03

**Authors:** Pankaj Khatiwada, Bian Yang, Jia-Chun Lin, Bernd Blobel

**Affiliations:** 1 Department of Information Security and Communication Technology (IIK), Norwegian University of Science and Technology (NTNU), 7034 Trondheim, Norway; bian.yang@ntnu.no (B.Y.); jia-chun.lin@ntnu.no (J.-C.L.); 2 Medical Faculty, University of Regensburg, 93053 Regensburg, Germany; bernd.blobel@klinik.uni-regensburg.de

**Keywords:** PGHD, health data, security, privacy, patient-generated health data

## Abstract

The evolution of Patient-Generated Health Data (PGHD) represents a major shift in healthcare, fueled by technological progress. The advent of PGHD, with technologies such as wearable devices and home monitoring systems, extends data collection beyond clinical environments, enabling continuous monitoring and patient engagement in their health management. Despite the growing prevalence of PGHD, there is a lack of clear understanding among stakeholders about its meaning, along with concerns about data security, privacy, and accuracy. This article aims to thoroughly review and clarify PGHD by examining its origins, types, technological foundations, and the challenges it faces, especially in terms of privacy and security regulations. The review emphasizes the role of PGHD in transforming healthcare through patient-centric approaches, their understanding, and personalized care, while also exploring emerging technologies and addressing data privacy and security issues, offering a comprehensive perspective on the current state and future directions of PGHD. The methodology employed for this review followed the Preferred Reporting Items for Systematic Reviews and Meta-Analyses (PRISMA) guidelines and Rayyan, AI-Powered Tool for Systematic Literature Reviews. This approach ensures a systematic and comprehensive coverage of the available literature on PGHD, focusing on the various aspects outlined in the objective. The review encompassed 36 peer-reviewed articles from various esteemed publishers and databases, reflecting a diverse range of methodologies, including interviews, regular articles, review articles, and empirical studies to address three RQs exploratory, impact assessment, and solution-oriented questions related to PGHD. Additionally, to address the future-oriented fourth RQ for PGHD not covered in the above review, we have incorporated existing domain knowledge articles. This inclusion aims to provide answers encompassing both basic and advanced security measures for PGHD, thereby enhancing the depth and scope of our analysis.

## 1. Introduction

This article is a completely revised and strongly extended version of our contribution to the pHealth 2022 Conference in Oslo [1]. In the predigital era, patient health data was mostly gathered during face-to-face consultation and manually recorded on paper. These records, mostly consisting of clinical observations, lab test data, physician notes, and imaging tests, were then archived as physical documents within healthcare facilities. The process had its shortcomings; not only was retrieving and disseminating these data cumbersome, but transferring such records between providers often meant mailing or faxing them, leading to potential delays or loss of vital information and security concerns. However, with technological advances in healthcare, there was a notable paradigm shift towards Electronic Health Records (EHRs) [2]. These digital systems streamlined the processes of storing, accessing, and sharing patient data. Concurrently, the healthcare sector has begun to employ standardized instruments and devices, allowing monitoring of specific health domains such as sleep or cardiac activity. This development is another key step towards the emergence of Patient-Generated Health Data (PGHD) [3]. With the advent of the digital era, patients have gained unprecedented access to an abundance of health information, leading to an increasing need for tools and platforms that enable monitoring of personal health metrics. The demand for better healthcare outcomes has been addressed by the advent of wearable devices and home-monitoring systems, which have provided valuable information outside the traditional boundaries of clinical settings [4]. Furthermore, the growing prevalence of digital platforms has improved the ease with which patients can record their daily experiences, including the onset of symptoms and the use of medications. The result of this is that doctors now have a more complete understanding of the health trajectory of their patients [5]. This transition has ramifications that extend beyond the scope of ordinary data collection. PGHD provides a holistic snapshot of a patient’s health, highlighting details that are sometimes missed during occasional clinical visits. The continuous nature of this data collection ensures real-time monitoring, facilitating prompt interventions should anomalies arise. In a more significant manner, the utilization of PGHD has effectively encouraged patients to assume a major role in shaping their health narratives, thus promoting increased participation and compliance with medical treatment plans [6]. Fundamentally, the shift from physical paper records to digital systems and the development of PGHD represent an enormous transformation. It shifted the focus from provider-centric periodic care to ongoing patient-driven support. Due to this transformation, preventive medicine, individualized treatment, and patient participation are now front and center in the healthcare system [7].

**Figure 1:**
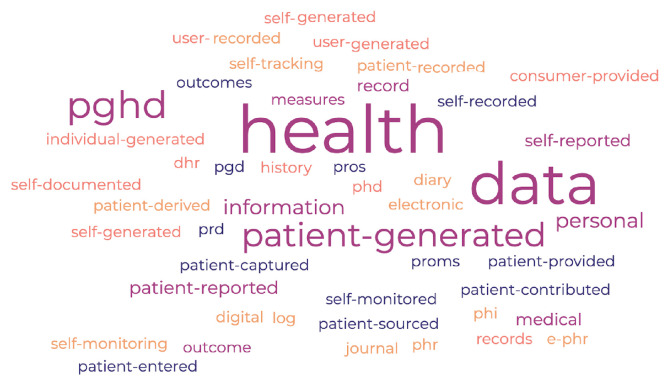
PGHD Related Word Cloud.

PGHD can be understood and expressed in different ways by researchers, healthcare professionals, and industry stakeholders. Figure 1 presents a word cloud that illustrates the various terms associated with Patient-Generated Health Data (PGHD). These may encompass, but are not limited to: Patient-Generated Health Data (PGHD), Patient-Reported Outcome Measures (PROMs), Patient-Reported Outcomes (PROs), Self-Reported Health Data, Patient-Reported Data (PRD), Self-Monitored Health Data, Self-Recorded Health Data, Patient-Entered Health Data, Patient-Captured Health Data, Patient-Generated Data (PGD), Patient-Provided Health Data, Patient-Sourced Health Data, Patient Contributed Health Data, Patient-Recorded Information, Patient Derived Health Data, Personal Health Record (PHR), Electronic Personal Health Record (e-PHR), Personal Health Information (PHI), Health Diary, Health Journal, Health Log, Self-Tracking Data, Health Self-Monitoring, Digital Health Records (DHR), Personal Medical History, User-Generated Health Data (UGH Data), Self-Reported Medical Information, Self-Generated Health Information, Self-Documented Health Data, Self-Entered Health Data, Individual-Generated Health Data, Consumer Provided Health Data, User-Entered Health Information, Personal Health Data (PHD). Throughout this article, we consistently use the term PGHD (Patient-Generated Health Data) to refer to health data produced by patients. Whenever the term health data, patient data, or generated data appears, it should be understood as referring specifically to PGHD.

The article is structured into seven sections. Section 2 provides the main objective of the article. Here, we will introduce various research questions (RQs), mainly organized into four categories, Exploratory, Impact Assessment, Solution-Oriented, and Future-Oriented RQs. These questions will be discussed in detail in the subsequent section of the article. In Section 3, we will shift our focus to a concise overview of PGHD, covering its definition and types. This section also explores the growth of PGHD driven by technological advances, identifies its potential stakeholders, and discusses the challenges associated with it. Additionally, it examines the applications of PGHD, its global adoption trends, and anticipates its future developments. Section 4 outlines the methodology used for conducting the review, addressing the research questions (RQs) from three perspectives: Exploratory, Impact Assessment, and Solution-Oriented RQs. Section 5 is dedicated to addressing the Research Questions (RQs) highlighted in Section 3, as identified from the review article in Section 4. This section also tackles Future-Oriented RQs, which, although not part of the review, but answered separately and independently. In Section 6, we discuss the findings and results from Section 5, followed by the final conclusions presented in Section 7.

## 2. Objectives

The primary purpose of the article is to conduct a review and comprehensively explore and synthesize the current understanding of PGHD in terms of its origin, types, technological underpinnings, stakeholders involved, challenges (with a keen focus on privacy, security, and regulatory challenges), benefits for personalized care, global perspectives, and future prospects. The review seeks to elucidate the significance of PGHD in revolutionizing healthcare delivery, emphasizing its role in improving patient-centric approaches, enabling personalized interventions, and identifying emerging technologies and practices. A section of this article will also look at the privacy and security concerns associated with PGHD, highlighting the evolving regulatory landscape in different areas and its implications on the broader adoption and utilization of PGHD. This review would provide stakeholders from healthcare providers to technology developers and policymakers with a holistic view of the current landscape of PGHD, its implications, and its potential trajectory in the foreseeable future.

PGHD is where technology, healthcare and the power of patients come together. As we try to understand all of its different parts, some key questions arise. These questions help guide our research and what we want to learn from this review. Grouped by their type and what they aim to find, these questions help point us to the important information we need. We categorized them into four sections as follows:

(a) Exploratory Questions: (PGHD Understanding and Patient Perspective)
  - RQE1: What are the primary sources of PGHD, and for what purpose are they being utilized in healthcare settings?
  - RQE2: How do patients perceive the collection and utilization of their generated PGHD?
(b) Impact Assessment Questions:
  - RQI1: What are the potential ramifications of data breaches pertaining to patient-generated health data on patient trust and healthcare outcomes?
  - RQI2: What impact does the incorporation of patients and their PGHD have on healthcare decision-making
(c) Solution-Oriented Questions:
  - RQS1: What are the recommended strategies for improving the security and privacy of PGHD?
  - RQS2: In what ways may healthcare providers and technology developers engage in collaborative efforts to achieve an ideal equilibrium between the value of PGHD and the safeguarding of data privacy and security?
(d) Future-Oriented Questions:
  - RQF1: How can PGHD prepare for the upcoming security and privacy problems brought on by the widespread adoption of AI and ML in healthcare?
  - RQF2: How may the next decade’s development of wearable technology and Internet of Medical Things devices affect the current state of PGHD security and privacy concerns?

Our approach will be to address the research questions from parts (a) Exploratory Questions (RQE1, RQE2), (b) Impact Assessment Questions (RQI1, RQI2), and (c) Solution-Oriented Questions (RQS1, RQS2) by leveraging the insights from the review conducted in Section 4. Regarding part (d) Future-oriented questions (RQF1, RQF2), due to the lack of extensive literature currently discussing these topics in PGHD, we will aim to provide answers by aligning them with the security of the Internet of Medical Things (IoMT) data and the security of health data, which are directly relatable to PGHD.

## 3. PGHD Understanding

This section focuses on providing the reader with a comprehensive overview of PGHD before discussing the RQs. It will differentiate PGHD from clinical data, discuss its various types, and explore its increasing prevalence. Additionally, it will identify key stakeholders involved, examine the challenges faced, and describe how PGHD is being used. The section will also dive into the adoption processes of PGHD and its ongoing advancements. As stated in the Introduction Section, PGHD refers to health-related data created, recorded, or gathered by or from patients (or family members or other caregivers) to address a health concern. This can include biometric data, symptoms, lifestyle choices, and other health-related information. PGHD is increasingly recognized for its potential to improve care and research, particularly in chronic disease management, mental health, and preventive care [2–20] [8]. Clinical data, on the other hand, refers to information collected by healthcare professionals in the course of providing care. This includes data from physical examinations, laboratory tests, imaging studies, and other diagnostic tools. Clinical data are typically considered more reliable because they are collected by professionals using standardized methods and equipment [9]. Below Table 1 provides the difference between the PGHD and clinical data:

### 3.1. PGHD Type

**Figure 2:**
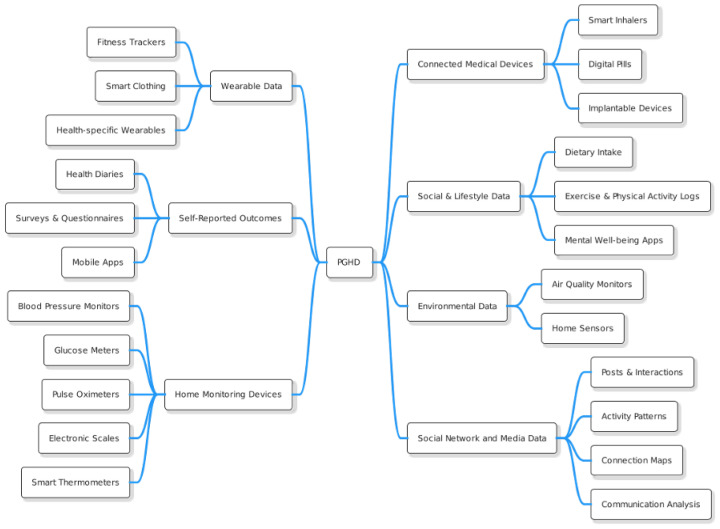
PGHD Types.

As we already know, PGHD refers to health-related data created, recorded, or collected by or from patients (or family members or other caregivers) to help address a health concern. These data are often outside the traditional clinical setting and provide insights that can contribute to a more comprehensive view of a patient’s health. The Figure 2 illustrates the different types of PGHD, with brief descriptions provided below.

#### 3.1.1. Wearable Data

- Fitness Trackers: Devices such as Fitbit, Garmin, or Apple Watch collect data on steps taken, heart rate, sleep patterns, calories burned, etc. [10].
- Health-specific Wearables: Devices that monitor specific health metrics, such as continuous glucose monitors for people with diabetes.
- Smart Clothing: Includes garments with embedded sensors that can monitor physiological markers such as heart rate, breathing rate, and muscle activity [11].

#### 3.1.2. Self-Reported Outcomes [12]

- Health Diaries: Patients may keep a daily or weekly record of symptoms, diet, medication intake, and other health-related factors.
- Surveys & Questionnaires: Tools such as the Patient-Reported Outcomes Measurement Information System (PROMIS) offer standardized measures of physical, mental, and social well-being.
- Mobile Apps: Many health and wellness apps allow users to input data related to mood, nutrition, menstrual cycles, pain levels, etc.

#### 3.1.3. Home Monitoring Devices [13]

- Blood Pressure Monitors: Devices that allow patients with hypertension or other conditions to monitor their blood pressure at home.
- Glucose Meters: Used by diabetics to regularly check blood sugar levels.
- Pulse Oximeters: Measure blood oxygen levels, which can be crucial for patients with respiratory conditions.
- Electronic Scales: For monitoring weight, especially useful for patients with heart failure or those undergoing certain treatments.
- Smart Thermometers: Digital devices that track and record temperature readings, often connecting to smartphone apps.

#### 3.1.4. Connected Medical Devices [14]

- Smart Inhalers: Help patients with asthma or COPD track medication use and offer reminders.
- Digital Pills: Pills embedded with edible sensors that send signals to external devices upon ingestion, ensuring medication adherence.
- Implantable Devices: Some devices, such as certain cardiac monitors, can transmit data to external receivers.

#### 3.1.5. Social & Lifestyle Data [15]

- Dietary Intake: Through apps or platforms where users input their daily meals and snacks.
- Exercise & Physical Activity Logs: Outside of wearables, users might manually record workouts or sports activities.
- Mental Well-being Apps: Tools like mood trackers or meditation apps can provide insight into mental health and stress levels.

#### 3.1.6. Environmental Data [16]

- Air Quality Monitors: Devices that measure air quality within or outside the home, which can be valuable for patients with allergies or respiratory conditions.
- Home Sensors: Detect things like mold, pollen, or other environmental factors that might affect health.

#### 3.1.7. Social Network and Media Data [17]

- Posts & Interactions: Analysis of posts, likes, comments, and shares on platforms like Facebook, Twitter, or Instagram can reveal emotional well-being, social support structures, and potential levels of stress or anxiety.
- Activity patterns: The time spent on social media platforms and the timing of activity can indicate sleep patterns and potentially correlate with mental health states.
- Connection Maps: Examination of the size and strength of a user’s social network can provide insights into social isolation or social well-being.
- Communication Analysis: Studying the content and frequency of messages or posts exchanged can help gauge the quality of social interactions and its impact on health.

While PGHD offers rich insights and complements clinical data, integrating and interpreting these data in clinical practice presents challenges. Healthcare providers must ensure the accuracy and relevance of the data, and systems must be in place to protect patient privacy and data security. However, as technology and interoperability improve, PGHD will increasingly play a pivotal role in personalized healthcare and proactive patient management.

### 3.2. PGHD Technological Rise

**Figure 3:**
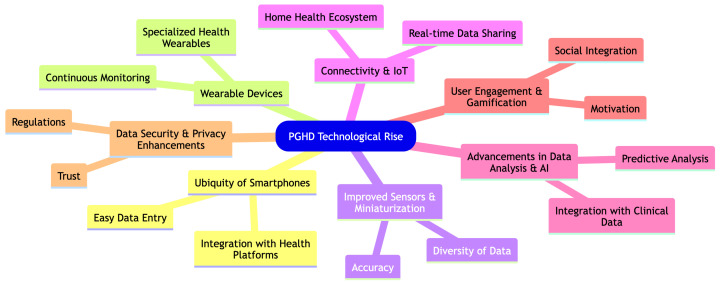
PGHD Technological Rise.

Technological advancements, particularly in the realms of mobile apps and wearable devices, have significantly facilitated the rise and importance of Patient-Generated Health Data (PGHD). Here is how these technological breakthroughs have influenced the trajectory of PGHD as shown in Figure 3.

#### 3.2.1. Ubiquity of Smartphones [18]

- Easy Data Entry: The widespread use of smartphones means that a large number of people have a powerful computer in their pocket, enabling them to easily input and track health data through specialized apps.
- Integration with Health Platforms: Operating systems like Apple’s iOS have integrated health platforms (e.g., Apple Health) that aggregate data from various health and fitness apps, creating a comprehensive health profile.

#### 3.2.2. Wearable Devices [10,11]

- Continuous Monitoring: Wearable devices such as smartwatches and fitness trackers monitor users’ health metrics continuously, capturing data such as heart rate, sleep patterns, and activity levels in real-time.
- Specialized Health Wearables: Beyond general fitness trackers, there are wearables tailored for specific conditions, such as continuous glucose monitors for diabetics, which offer real-time insights and alerts.

#### 3.2.3. Improved Sensors & Miniaturization [19]

- Accuracy: Advancements in sensor technology mean that wearables and mobile devices can capture health data with increased accuracy, making the data more clinically relevant.
- Diversity of Data: From tracking UV exposure to measuring electrodermal activity (a potential stress indicator), technological progress has enabled a wider range of health metrics to be monitored.

#### 3.2.4. Connectivity & IoT (Internet of Things) [20]

- Real-Time Data Sharing: Devices can instantly upload data to the cloud, allowing real-time sharing with healthcare providers or integration with electronic health records (EHRs).
- Home Health Ecosystem: Smart home devices, such as connected scales or blood pressure monitors, can now seamlessly integrate with other health devices and platforms.

#### 3.2.5. Advancements in Data Analysis & AI [21]

- Predictive Analysis: With a large amount of PGHD being generated, advanced analytics and AI can identify patterns, predict potential health problems, and offer personalized health recommendations.
- Integration with Clinical Data: Advanced platforms can now integrate PGHD with traditional clinical data, offering healthcare providers a more holistic view of a patient’s health.

#### 3.2.6. User Engagement & Gamification [22]

- Motivation: Many health apps and wearables incorporate gamification elements, motivating users to achieve health goals, complete challenges, or maintain streaks, thus encouraging consistent data generation.
- Social Integration: Sharing achievements, joining fitness groups, or participating in community challenges can boost engagement and data generation.

#### 3.2.7. Data Security & Privacy Enhancements [23]

- Trust: As technology companies prioritize data security and privacy, users are more inclined to share and store their health data.
- Regulations: Advances in technology have been complemented by regulations, ensuring that PGHD is handled with the same care and security as traditional health data.

In conclusion, the confluence of advances in mobile technology, wearables, connectivity, and data analysis has transformed PGHD from a niche concept to a central player in modern healthcare. The real-time, continuous, and diverse nature of the data captured has enormous potential to revolutionize patient care, drive proactive health management, and contribute to personalized medicine.

### 3.3. PGHD Stakeholders

**Figure 4:**
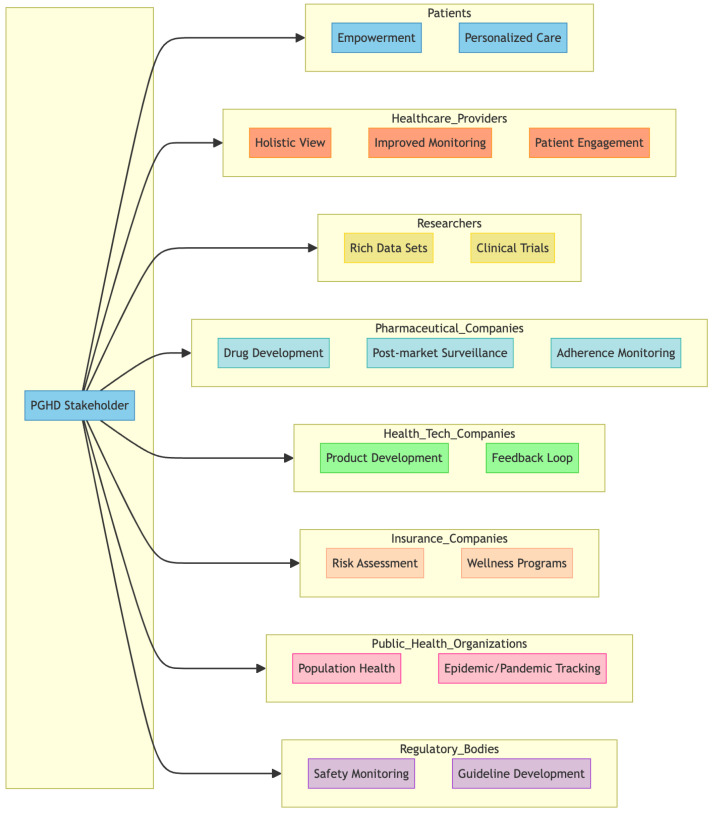
PGHD Stakeholders.

PGHD has value for a wide range of stakeholders [7,24,25] within the healthcare ecosystem. Figure 4 represents a breakdown of some of the primary stakeholders and how they are impacted by or use PGHD.

#### 3.3.1. Patients

- Empowerment: PGHD allows patients to play a more active role in their health management, giving them insight into their health trends and patterns.
- Personalized care: With a more comprehensive data profile, patients can receive more personalized health advice and treatments.

#### 3.3.2. Healthcare Providers

- Holistic View: PGHD complements clinical data, providing a more comprehensive picture of the health of the patient, including lifestyle and environmental factors.
- Improved monitoring: Especially for chronic conditions, PGHD enables providers to track patient health status in real time, allowing timely interventions.
- Patient Engagement: PGHD tools can foster better communication between patients and providers, improving adherence to care plans.

#### 3.3.3. Researchers

- Rich Data Sets: With vast amounts of PGHD generated, researchers have access to diverse real-world data that can provide insight into health trends, disease patterns, and treatment outcomes.
- Clinical Trials: PGHD can be used to monitor participants in clinical trials, providing real-time data and potentially reducing costs.

#### 3.3.4. Pharmaceutical Companies

- Drug Development: PGHD can provide information on how patients respond to medications in real-world settings, which can inform drug development and optimization.
- Post-market surveillance: After a drug is released, PGHD can help track its efficacy and any potential side effects in the broader population.
- Adherence Monitoring: Companies can understand how often patients take their medications and the factors that influence adherence.

#### 3.3.5. Health Tech Companies

- Product Development: Companies can use PGHD to develop new devices, apps, and platforms tailored to user health needs.
- Feedback Loop: Continuous input from PGHD allows tech companies to refine and optimize their health tech offerings.

#### 3.3.6. Insurance Companies

- Risk Assessment: PGHD can provide information on an individual’s health habits, potentially influencing underwriting and policy pricing.
- Wellness Programs: Many insurers now offer wellness programs that leverage PGHD to incentivize healthy behaviors, potentially reducing claims in the long run.

#### 3.3.7. Public Health Organizations

- Population Health: PGHD can offer information on health trends at the community or population level, helping to plan health campaigns and allocate resources.
- Epidemic/Pandemic Tracking: In situations such as the COVID-19 pandemic, PGHD from wearables or symptom-tracking apps can assist in early detection and monitoring of disease spread.

#### 3.3.8. Regulatory Bodies

- Safety Monitoring: PGHD can be a source of data to monitor the safety and efficacy of medical devices or treatments in real world settings.
- Guideline development: Real-world data from PGHD can inform the development of health guidelines and standards.

As healthcare becomes more patient-centric and data-driven, the relevance of PGHD is set to grow. Each stakeholder in the healthcare ecosystem can harness the potential of PGHD to improve outcomes, optimize resources, and drive innovation. However, collaboration among these stakeholders is crucial to ensure that PGHD is used effectively and ethically.

### 3.4. PGHD Challenges

**Figure 5:**
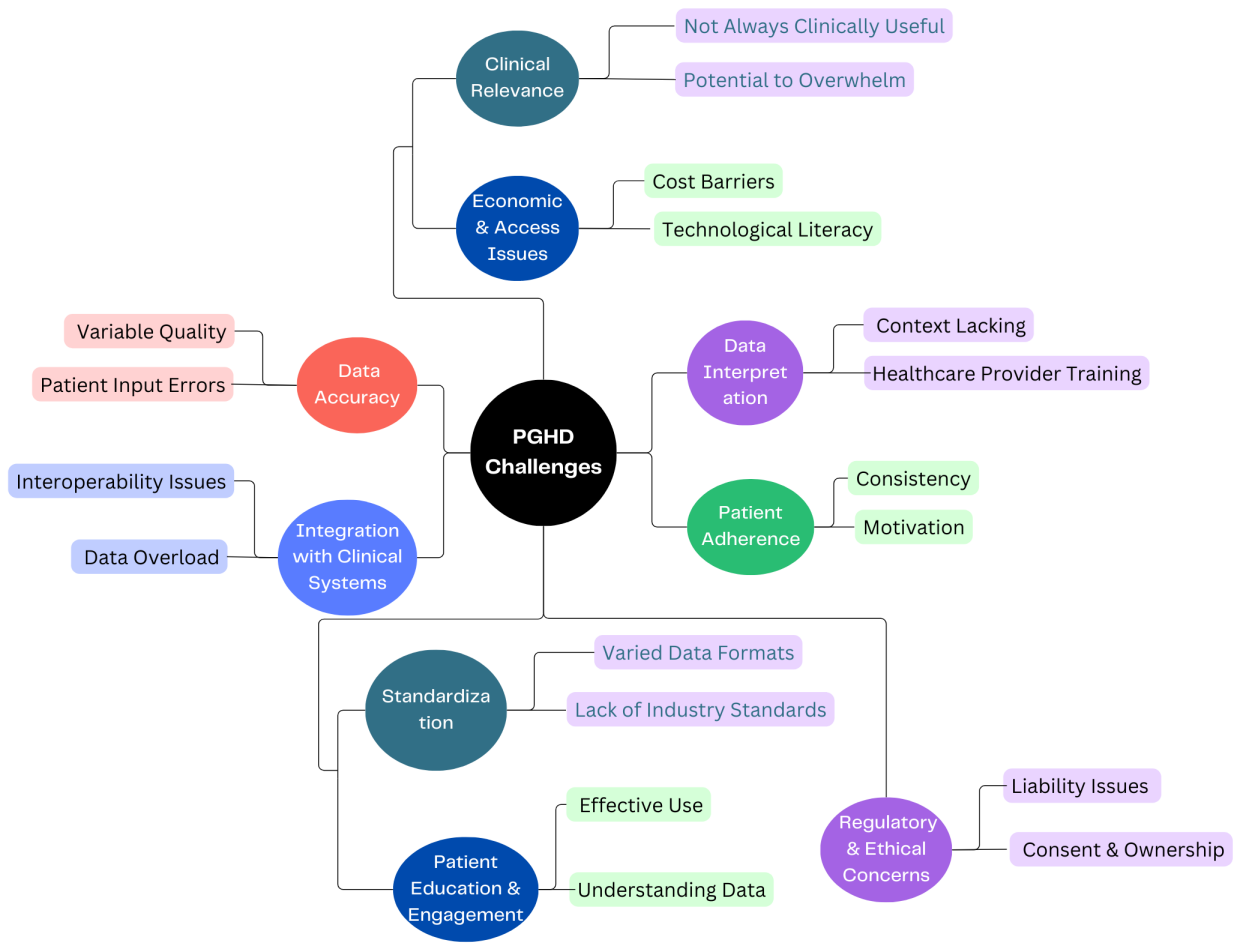
PGHD Challenges.

Certainly, while data security and privacy are the main concerns of PGHD, there are several other challenges associated with its use and implementation, as shown in Figure 5.

#### 3.4.1. Data Accuracy [26]

- Variable Quality: Devices and apps vary in their accuracy and reliability. For instance, one fitness tracker might measure steps or heart rate differently from another.
- Patient Input Errors: Manual data entries by patients, such as symptom logs or dietary intakes, can be prone to inaccuracies or inconsistencies.

#### 3.4.2. Integration with Clinical Systems [27]

- Interoperability Issues: Clinical systems like EHRs might not easily integrate with PGHD sources, leading to fragmented data.
- Data Overload: The sheer volume of PGHD can be overwhelming. Healthcare providers need efficient systems to sift through and extract relevant insights without being inundated.

#### 3.4.3. Patient Adherence [28]

- Consistency: Patients might not consistently use wearables or input data into health apps, medication management leading to gaps in data.
- Motivation: Maintaining motivation to regularly input data or use health-tracking tools can wane over time, especially if patients do not see immediate benefits.

#### 3.4.4. Data Interpretation [29]

- Context Lacking: Without proper context, PGHD can be misleading. For example, a spike in heart rate might be due to exercise, stress, or a health anomaly.
- Healthcare Provider Training: Not all providers may be trained or feel comfortable interpreting PGHD, especially given its diverse sources.

#### 3.4.5. Standardization [29]

- Varied Data Formats: Data from different devices or apps might come in a variety of formats, making aggregation and analysis challenging.
- Lack of Industry Standards: Without universally accepted standards for the collection and interpretation of PGHD, its clinical utility can be limited.

#### 3.4.6. Patient Education & Engagement [30]

- Effective Use: Patients need to be educated on how to effectively use devices or apps to ensure the data collected is of value.
- Understanding Data: Patients may misinterpret their data, leading to unnecessary anxiety or incorrect self-diagnosis.

#### 3.4.7. Clinical Relevance

- Not Always Clinically Useful: Although PGHD can offer many insights, not all of it may be relevant for clinical decisions [31].
- Potential to Overwhelm: Excessive data can lead to “alert fatigue”, in which healthcare providers become desensitized to numerous alerts or notifications, potentially overlooking important ones [32].

#### 3.4.8. Economic & Access Issues [33]

- Cost Barriers: Not all patients can afford smart wearables or devices, potentially leading to disparities in who can benefit from PGHD.
- Technological literacy: Not everyone is tech-savvy, and some may find it challenging to navigate health apps or devices.

#### 3.4.9. Regulatory & Ethical Concerns [34]

- Liability Issues: If a patient’s PGHD indicates a health issue but is not acted on, it raises questions about liability.
- Consent & Ownership: Clear guidelines on who owns PGHD and how it can be used are essential to navigate potential ethical dilemmas. Ethical concerns in healthcare such as privacy, consent, and data security are increasingly significant. As a response, regulations are evolving to safeguard personal health data and mitigate associated risks. This evolution might lead to the development of new regulations encompassing a broader range of health data sources. Consequently, technology companies could face more stringent laws related to health data. Additionally, there need to be novel approaches introduced for individuals to consent to the use of their health data in research.

Addressing these challenges requires collaboration between technology developers, healthcare providers, regulators, and patients. As the field matures, solutions to many of these challenges are likely to emerge, paving the way for PGHD to realize its full potential in healthcare.

### 3.5. PGHD Health Use

**Figure 6:**
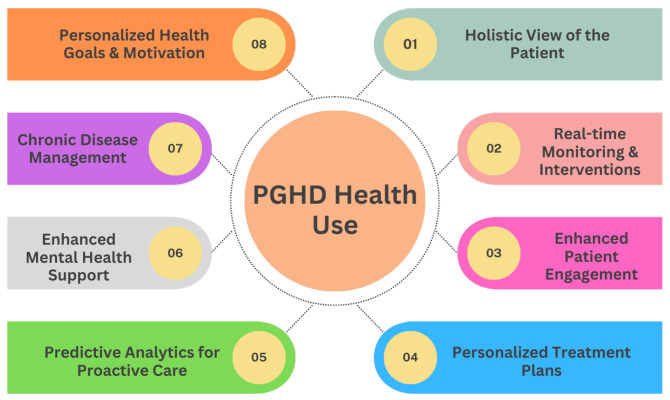
PGHD Health Use.

PGHD stands as transformative data in the shift toward personalized medicine, fundamentally reshaping the patient-clinician relationship and the very nature of healthcare delivery. Figure 6 provides eight various uses of PGHD in promoting health and a more patient-centric approach, enabling personalized care.

#### 3.5.1. Holistic View of the Patient [7]

- Comprehensive Data: PGHD captures aspects of daily life, such as diet, exercise, stress levels, and sleep patterns. When combined with clinical data, providers get a fuller picture of a patient’s health status and lifestyle.
- Environmental and Behavioral Context: Beyond just symptoms and clinical results, PGHD offers insights into the environments and behaviors affecting a patient’s health.

#### 3.5.2. Real-Time Monitoring & Interventions [29]

- Immediate Feedback: Devices that provide real-time data allow timely interventions. For example, a sudden drop in blood sugar levels captured by a continuous glucose monitor can trigger an immediate alert to a diabetic patient.
- Adjusting Treatment in Real-Time: Regular input of PGHD can help clinicians adjust medication dosages, exercise routines, or other treatment modalities based on current and actual data rather than waiting for periodic check-ups.

#### 3.5.3. Enhanced Patient Engagement [35]

- Active Participation: By tracking and sharing their own data, patients become active participants in their care journey.
- Educated Decisions: Access to their data empowers patients with knowledge, enabling them to make informed decisions about their health and engage in meaningful discussions with healthcare providers.

#### 3.5.4. Personalized Treatment Plans [36]

- Tailored Interventions: With PGHD insights, clinicians can develop care plans that are more aligned with a patient’s unique circumstances, whether it is customizing a physical therapy routine or dietary recommendations.
- Drug Response Monitoring: By capturing how a patient feels or reacts after taking the medication, PGHD can help personalize drug regimens to maximize efficacy and minimize side effects.

#### 3.5.5. Predictive Analytics for Proactive Care [37]

- Anticipating Health Issues: With the help of AI and machine learning, PGHD can be used to identify patterns and predict potential health problems before they become serious.
- Risk stratification: PGHD can aid in determining which patients are at higher risk for certain complications or conditions, enabling preemptive interventions.

#### 3.5.6. Enhanced Mental Health Support [36]

- Emotional Well-being Tracking: Apps that track mood or mental well-being can inform interventions, helping providers understand triggers and patterns in mental health fluctuations.
- Personalized Therapeutic Interventions: Mental health practitioners can use PGHD to tailor therapeutic strategies, such as recommending specific stress reduction techniques based on tracked stressors.

#### 3.5.7. Chronic Disease Management [38]

- Self-management: For chronic diseases such as diabetes or hypertension, PGHD allows patients to self-manage more effectively, adjusting behaviors in real time based on feedback from devices.
- Telehealth Integration: PGHD can be seamlessly integrated into telehealth platforms, allowing clinicians to provide remote care based on actual patient-generated metrics.

#### 3.5.8. Personalized Health Goals & Motivation [39]

- Setting Achievable Targets: With the granularity of PGHD, patients can set and work toward specific, personalized health goals, whether it is achieving a certain activity level or maintaining a dietary regimen.
- Gamification and Incentives: Many health apps use gamification elements, providing rewards or achievements based on individual user data, thus motivating consistent healthy behaviors.

In summary, PGHD shifts the paradigm from a generalized, reactive healthcare model to a personalized, proactive one. With the individual at the center of the care model, interventions are expedited, treatments are more aligned with personal needs, and the entire healthcare experience is more collaborative and effective.

### 3.6. PGHD Adoption Worldwide

The adoption and utilization of PGHD vary significantly throughout the world, influenced by a combination of technological infrastructure, regulatory environments, cultural attitudes, and economic factors. Here is a brief look at the global perspective on PGHD:

#### 3.6.1. North America (Primarily USA and Canada) [23,40,41]

- Advanced Adoption: The region has seen substantial growth in the adoption of wearable devices and health applications, supported by a robust technological infrastructure and a strong focus on healthcare innovation.
- Regulatory environment: Regulatory bodies like the FDA in the US provide guidelines for health applications and wearables, ensuring safety and efficacy.
- Challenges: Cost and insurance coverage can be barriers. Privacy concerns, especially in the US with regulations like HIPAA, also influence the utilization of PGHD.

#### 3.6.2. Europe [23,42]

- Varied Adoption: Northern and Western European countries, such as the UK, Germany and Scandinavia, are at the forefront of PGHD adoption, with a strong emphasis on digital health and e-health strategies.
- Data Protection: The General Data Protection Regulation (GDPR) sets stringent standards for data privacy, affecting how PGHD is collected and used.
- Cultural Openness: In general, there is a positive attitude towards using technology to enhance healthcare, although individual perceptions can vary.

#### 3.6.3. Asia [43,44,45]

- Rapid Growth: Countries such as Japan, South Korea, and Singapore are quickly adopting PGHD tools, driven by technological advancements and aging populations.
- Emerging markets: In India and China, the growth of the middle class and increasing tech-savviness are driving interest in personal health tracking, although full integration into healthcare systems is still in progress.
- Cultural Barriers: In certain areas, traditional beliefs about health can influence the acceptance and trust of digital health tools.

#### 3.6.4. Australia & New Zealand [46]

- Positive Adoption: Both countries are progressively integrating PGHD into their healthcare systems, supported by national e-health strategies and initiatives.
- Challenges Geographic dispersion, especially in Australia, can pose challenges for consistent PGHD adoption across urban and rural areas.

#### 3.6.5. Africa [47,48,49]

- Infrastructural Challenges: Limited technological infrastructure in many parts of the continent poses challenges to widespread adoption of PGHD.
- Innovative Solutions: Mobile phones are widely used in Africa, and there are health initiatives that leverage mobile technology for data collection, especially for community health.
- Cultural Differences Acceptance of PGHD varies, with some regions showing skepticism towards digital health interventions, while others are more receptive.

#### 3.6.6. Latin America [50,51]

- Growing Interest: Urban centers in countries such as Brazil, Argentina, and Mexico are showing increasing interest in wearable devices and health apps.
- Infrastructural Limitations: Inconsistent access to the high-speed internet and advanced medical technology can limit the integration of PGHD.
- Cultural views: While there is general openness to PGHD in many areas, trust in technology and data privacy concerns can vary widely.

In conclusion, while the potential of PGHD is recognized globally, its actual adoption and utilization are heavily influenced by regional factors. Advanced technological infrastructure, supportive regulations, economic capabilities, and cultural beliefs all play a role in how different parts of the world approach and integrate PGHD into their healthcare systems.

### 3.7. PGHD Advancement

**Figure 7:**
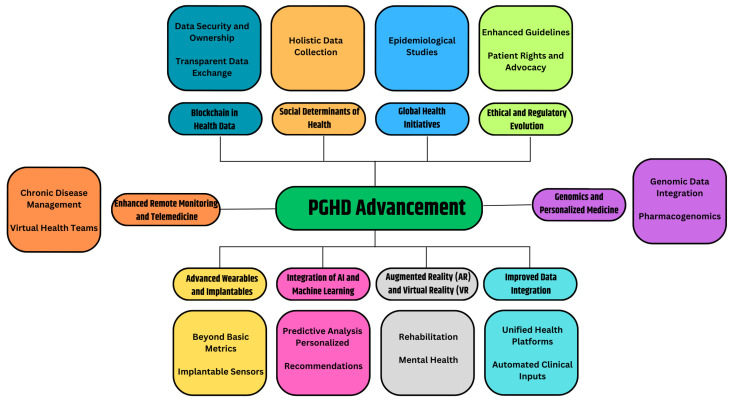
PGHD Advancement.

Certainly, the realm of PGHD is poised for continued growth and evolution. As technologies advance and healthcare evolves further towards patient-centric and preventive models, the role of PGHD will only become more central. Figure 7 provides the prospective future of the advancement of PGHD in healthcare.

#### 3.7.1. Advanced Wearables and Implantable [52]

- Beyond Basic Metrics: Future wearables will capture more than just heart rate or steps. They could monitor hydration levels, nutritional intake, stress biomarkers, or even blood oxygen levels in real-time.
- Implantable Sensors: Imagine tiny devices implanted under the skin or inside the body, continuously monitoring specific health parameters and sending alerts or recommendations when necessary.

#### 3.7.2. Integration of AI and Machine Learning [53,54]

- Predictive Analysis: By analyzing vast amounts of PGHD, AI systems can identify patterns and predict health events or complications even before they manifest themselves overtly.
- Personalized recommendations: AI-driven apps could offer users daily health, diet, or exercise recommendations based on real-time data.

#### 3.7.3. Augmented Reality (AR) and Virtual Reality (VR) [55,56]

- Rehabilitation: VR setups can be used for physical or cognitive therapy, with PGHD tracking progress and adjusting routines.
- Mental health: AR/VR environments can be therapeutic, especially when combined with real-time biofeedback.

#### 3.7.4. Improved Data Integration [57,58]

- Unified Health Platforms: A consolidated platform where clinical data and PGHD converge, allowing seamless communication between healthcare providers and patients.
- Automated Clinical Inputs: Devices that not only collect data, but can also automatically input this into Electronic Health Records (EHRs) without manual intervention.

#### 3.7.5. Genomics and Personalized Medicine [59,60]

- Genomic Data Integration: The decreasing cost of genome sequencing has facilitated the integration of genetic data with PGHD, allowing valuable insights into individual predispositions and the customization of preventive treatments.
- Pharmacogenomics: Personal drug regimens based on genetic makeup, lifestyle, and real-time health data.

#### 3.7.6. Enhanced Remote Monitoring and Telemedicine [61]

- Chronic Disease Management: Real-time PGHD can be transmitted to healthcare providers, allowing them to monitor and manage chronic conditions from a distance.
- Virtual Health Teams: Based on PGHD inputs, virtual multidisciplinary teams can convene and make collaborative decisions.

#### 3.7.7. Blockchain in Health Data [62]

- Data Security and Ownership: Blockchain can provide decentralized, immutable ledgers for health data, ensuring security and giving patients more control and ownership of their data.
- Transparent Data Exchange: Facilitating trust in the sharing of PGHD among patients, providers, and researchers.

#### 3.7.8. Social Determinants of Health [63]

- Holistic Data Collection: Beyond biological parameters, future PGHD tools might capture data on social determinants such as environment, economic conditions, or social interactions, offering a comprehensive view of factors affecting health.

#### 3.7.9. Global Health Initiatives [64]

- Epidemiological Studies: The large-scale collection of PGHD can help track disease outbreaks, understand public health trends, and shape health policies.

#### 3.7.10. Ethical and Regulatory Evolution [23]

- Enhanced Guidelines: As PGHD becomes central to healthcare care, regulatory bodies will provide clearer guidelines to ensure data accuracy, privacy, and appropriate use.
- Patient Rights and Advocacy: Enhanced focus on patients’ rights to their data, how it is used, and who has access.

In essence, the future of PGHD is a confluence of technology, personalized medicine, and patient empowerment. As innovations continue to emerge, the boundary between traditional healthcare settings and everyday life will blur, making health management an integrated aspect of our daily routines.

## 4. Methodology

The systematic review was carried out according to the Preferred Reporting Items for Systematic Reviews and Meta-Analyses (PRISMA) guidelines [65].

### 4.1. Search Strategy

To comprehensively review the existing literature on PGHD with a specific emphasis on its understanding, use, patient prospective, future trends and security and privacy, a systematic search strategy was formulated. The strategy was carefully tailored to each database, given its specificities and nuances.

Database Utilized

- PubMed
- Scopus
- IEEE Xplore
- Web of Science
- ACM Digital Library
- EBSCO host

### 4.2. Search Terms and Boolean Operators

- Patient Data Terms: Patient-Generated Health Data, PGHD, Patient-reported outcomes, Personal health records
- Security and Privacy Terms: security, privacy, encryption, health data security, health data privacy

The terms within each thematic group were combined using the “OR” Boolean operator, ensuring the breadth of the search within that theme. The two primary themes (Patient Data and Security & Privacy) were combined using the “AND” Boolean operator to ensure that the results pertain to both aspects.

Key Words Used: (“Patient-generated health data” OR “PGHD” OR “Patient-reported outcomes” OR “Personal health records” OR “PHR”) AND (“security” OR “privacy” or “encryption” or “health data security” OR “health data privacy”)

Search strategies for individual databases:

#### 4.2.1. Pubmed

For the topic “patient-generated health data security and privacy” and focusing specifically on PubMed, it is recommend using a combination of MeSH (Medical Subject Headings) terms and free-text keywords. Search terms can be organized into thematic groups and combined using Boolean operators. (“Patient-generated health data” [MeSH] OR “PGHD” OR “Patient-reported outcomes” OR “Personal health records” OR “PHR”) AND (“security” OR “privacy” or “encryption” or “health data security” OR “health data privacy”).

#### 4.2.2. Scopus

When designing a search strategy for Scopus, it is important to consider the database’s specific syntax and features. Scopus does not use MeSH terms like PubMed; instead, you will focus on keyword and free-text searching.

TITLE-ABS-KEY (“Patient-generated health data” OR “PGHD” OR “Patient-reported outcomes” OR “Personal health records” OR “PHR”) AND TITLE-ABS-KEY (“security” OR “privacy” OR “encryption” OR “health data security” OR “health data privacy”)

In this query:

TITLE-ABS-KEY ensures that the search is focused on the titles, abstracts, and keywords of the documents, which are likely where the most relevant information will be.

We use parentheses to group related terms and use the Boolean operators AND and OR to specify our search requirements.

#### 4.2.3. IEEE Xplore

IEEE Xplore is a platform that mainly contains literature in the fields of electronics, electrical engineering, and computer science. Thus, when designing a search strategy for IEEE Xplore, we should consider its audience and the likelihood that our terms will pull relevant results.

(“Patient-generated health data” OR “PGHD” OR “Patient-reported outcomes” OR “Personal health records” OR “PHR”) AND (“security” OR “privacy” or “encryption” or “health data security” OR “health data privacy”)

Keep the following in mind when searching on IEEE Xplore:

Given the technical nature of the database, you are likely to encounter articles that deal with the technical aspects of PGHD security and privacy. This could be beneficial if you are interested in the technical solutions and challenges of PGHD.

It is crucial to review the results for relevance, as IEEE Xplore’s focus on engineering and technology may yield some results that are tangential to the primary health-focused aspect of PGHD.

#### 4.2.4. Web of Science

Web of Science (WoS) is a multidisciplinary database, which means that it is essential to have a well-structured search strategy to yield the most relevant results. Given your focus on “patient-generated health data” (PGHD) with an emphasis on security and privacy, here is a proposed search strategy for WoS:

TS = (“Patient-generated health data” OR “PGHD” OR “Patient-reported outcomes” OR “Personal health records” OR “PHR”) AND TS = (“security” OR “privacy” OR “encryption” OR “health data security” OR “health data privacy”)

In this search query:

TS = specifies that the search should look at topic, which includes title, abstract, author keywords, and Keywords Plus in WoS.

The terms within each group are combined using the OR operator to broaden the search within that theme.

The two main themes (PGHD and Security & Privacy concepts) are combined using the AND operator to ensure the results have elements of both.

#### 4.2.5. ACM Library

When constructing a search strategy for the ACM Digital Library, remember that it mainly caters to the disciplines of computing and technology. Given your focus on “patient-generated health data” (PGHD) and its aspects of security and privacy, here is a possible search strategy for the ACM Digital Library:

[[All: “patient-generated health data”] OR [All: “pghd”] OR [All: “patient-reported outcomes”] OR [All: “personal health records”] OR [All: “phr”]] AND [[All: “security”] OR [All: “privacy” or “encryption” or] OR [All: “health data security”] OR [All: “health data privacy”]].

#### 4.2.6. EBOSCO Host

EBSCOhost provides a variety of databases with a broad range of disciplines, so it is essential to ensure your search strategy is well-structured to yield relevant results.

(“Patient-generated health data” OR “PGHD” OR “Patient-reported outcomes” OR “Personal health records” OR “PHR”) AND (“security” OR “privacy” or “encryption” or “health data security” OR “health data privacy”).

### 4.3. Inclusion and Exclusion Criteria for Studies

The inclusion and exclusion criteria are imperative to move through the results and ensure that the review captures the most relevant studies.

Inclusion Criteria:

- Articles that discuss or analyze patient-generated health data.
- Peer-reviewed articles, journal papers, conference papers, or books on PGHD.
- Articles published in English.
- Articles indexed in Google Scholar, SCOPUS, SCI, or SCIE.
- Both qualitative (primary and secondary research) and quantitative studies were included.
- Accessible through the university library and open access.
- Studies focusing on PGHD in the context of health data security.

Exclusion Criteria:

- Articles that only peripherally mention patient-generated health data without in-depth analysis or discussion.
- Articles not available in full text.
- Articles not in English or peer reviewed on PGHD.
- Poster, editorial and commentary papers excluded.
- Research mainly focused on extensive clinical data as opposed to PGHD.

### 4.4. Search Results

The search was conducted on 12 September 2023, and the initial results for each database were: Scopus = 1301, IEEE Xplore = 207, Web of Science = 703, PubMed. = 621, ACM Digital Library = 408, EBSCO Host = 242. Following the application of the inclusion and exclusion criteria, these numbers will be further refined to arrive at the final set of articles for review.

### 4.5. Study Selection and Screening Process

We used Rayyan, an online tool for systematic reviews, to collaboratively screen potential articles. First, Rayyan goes through the entries to eliminate duplicates. After that, the articles were shortlisted based on the review of their titles and abstracts. We then evaluated the full text of these articles using inclusion and exclusion criteria to determine their suitability for our systematic review. Any disagreements were addressed by team discussions until a mutual agreement was reached.

**Figure 8:**
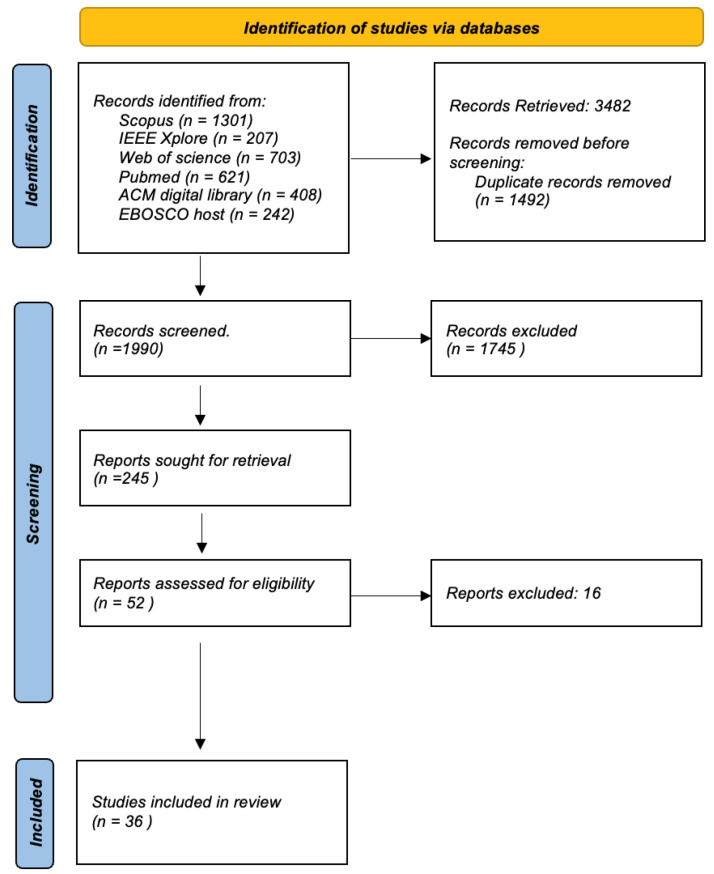
Selection Process.

The Figure 8 shows a flow chart detailing the systematic review process for the selection of articles from various academic databases. It starts with the identification of studies, where a total of 3482 records were retrieved from databases such as Scopus (1301 records), IEEE Xplore (207 records), Web of Science (703 records), PubMed. (621 records), ACM Digital Library (408 records) and EBOSCO host (242 records). Before screening, duplicate records were removed, which amounted to 1492, narrowing the field to 1990 unique records that were screened for relevance using inclusion and exclusion criteria as explained above. From these, 245 reports were identified as potentially relevant and were retrieved for further assessment. Of these, a deeper evaluation was done on 52 reports to determine their eligibility based on predefined criteria relevant to the review’s focus. This led to the exclusion of 20 reports that likely did not meet inclusion criteria such as study design, relevance to the research question, or quality thresholds. The final inclusion saw 32 studies that passed all previous filters and were deemed suitable for the review. These studies will be thoroughly reviewed and analyzed to answer the research question at hand. The flow chart provides a clear and structured outline of the literature selection process, ensuring a rigorous and transparent review method. This process is essential to minimize bias and ensure that the review is comprehensive and based on the best available evidence.

### 4.6. Study Selection and Study Characteristics

The comprehensive review of the literature in this field yielded a total of 36 peer-reviewed articles eligible for citation, spanning several esteemed publishers and databases. These include 17 articles from PubMed, 5 from Scopus, 7 from Web of Science, 3 from IEEE Xplore, and 4 from ACM Library. The articles predominantly originated in the United States, with a considerable number also coming from various other countries throughout Europe, Asia, Australia, and North America. In terms of research type, the articles demonstrated a diverse range of methodologies: 15 were categorized as interviews, 16 as regular articles, 4 as review articles, and 1 as a comment paper. Furthermore, the research encompassed various study formats, including a survey, an empirical study, a Delphi study, and a unique blend of interviews and implementation. Geographically, the studies spanned multiple continents with the following detailed distribution: USA (n = 25), Australia (n = 3), UK (n = 2), Ireland (n = 1), Switzerland (n = 1), Canada (n = 1), Iraq (n = 1), India (n = 1), Slovenia (n = 1), Pakistan (n = 2), Sweden (n = 1), South Korea (n = 1), Netherlands (n = 1) and China (n = 1). This global representation highlights the widespread interest and applicability of the research topic.

The selected studies were categorized into various groups, providing insights into different aspects of the research topic. These categories included interviews (n = 15), regular articles (n = 16), review articles (n = 4), and other formats such as comment articles and empirical studies. This classification underscores the multifaceted nature of research and the diverse methodologies employed in exploring the field.

As mentioned previously, the first three research questions (RQs) (a) Exploratory Questions (RQE1, RQE2), (b) impact assessment questions (RQI1, RQI2) and (c) solution-oriented questions (RQS1, RQS2) are addressed by the articles in the review, as depicted in the Table 2.

Table 3 details the articles that contribute to the understanding of basic and advanced security measures in the PGHD domain, drawing on existing security techniques and protocols in related health sectors, such as medical device data security and health data security and privacy. These additional articles have been instrumental in addressing RQs (RQF1 and RQF2), compensating for the scarcity of literature specifically focused on the PGHD domain.

## 5. Result

The findings related to the identified RQs are elaborated as follows.

### 5.1. RQE1: What Are the Primary Sources of PGHD, and for What Purpose Are They Being Utilized in Healthcare Settings?

Kawu et al. [27] identified the primary sources of PGHD used in healthcare settings as: Mobile health apps and wearable devices like fitness trackers, these collect various clinical measurements like heart rate, blood pressure, sleep data, etc. They are used for monitoring chronic conditions, weight management, fitness tracking, etc. Patient portals and personal health records (PHRs)—These allow patients to directly enter health data like symptoms, mood, food intake, pain levels, etc. Used for self-monitoring and sharing with providers. Social media posts—Posts describing health experiences, symptoms, etc. can provide insights especially for public health monitoring. Sensors and Internet-of-Things (IoT) devices—Various sensors and networked devices can transmit health data like ECG, glucose levels, etc. Used for remote monitoring. Interactive voice response systems—Allow patients to provide symptom or medication data via voice commands. Help track treatment progress. Virtual/mixed reality systems—Can be used for physical therapy, pain management, behavioral health, etc. The main purposes for using these sources of PGHD are: (1) To improve patient self-management of chronic conditions (2) Allow remote monitoring and care outside clinics (3) Provide a more holistic view of health by capturing patient experiences and lifestyle (4) Reduce burden on the healthcare system via automated data collection (5) Enable personalized care through additional patient context (6) Promote patient participation and empowerment in their own healthcare. However, the review mentions that more research is needed to incorporate emerging sources of PGHD like social networks, virtual reality, etc. into clinical workflows and health IT systems. Concerns around data privacy, quality, and interoperability also need to be addressed.

Petersen et al. [34] highlighted that PGHD encompasses a variety of sources that are increasingly being integrated into healthcare settings to improve patient care and outcomes. These sources include patient-reported outcomes (PRO), which assess how patients perceive their health status; patient-powered patient registries (PPR) that collect standardized information about specific patient groups; patient-powered research networks (PPRN) that consolidate multiple registries; and patient portals that offer web or mobile access to provider-created health information. In addition, remote sensors and smart wearable devices are becoming prevalent, transmitting personal data such as heart rate and activity levels for external analysis. The digital age has also seen the rise of social media platforms and mobile health (mHealth) apps as tools for patients to track, record, or share their health data. Integrating PGHD into healthcare care aims to improve care, facilitate research, assess treatment effectiveness, provide patients with access to their health information and foster better communication among patients, caregivers and healthcare providers. In essence, leveraging PGHD from a variety of sources provides a comprehensive view of patient experiences, making it possible to tailor care, expand research, and improve overall health outcomes.

Lai et al. [66] explain that PGHD comes from a variety of consumer health technologies including smartphones, wearable devices, sensors, patient portals, and personal health records. Patients can use these technologies to track health data such as vital signs, symptoms, lifestyle behaviors, and patient-reported outcomes. This data is being used in several ways. Patients are using PGHD for self-monitoring and self-management of health conditions, especially chronic diseases. PGHD also allows new methods to capture patient perspectives and outcomes in clinical research, such as clinical trials. In addition, PGHD is being integrated into electronic health records and provider workflows to inform clinical decision-making and care delivery, giving providers a more comprehensive view of patients. PGHD analysis from sources such as mobile health apps and online communities provides insights into patients’ experiences, behaviors, and needs, guiding the design of better tools and interventions. In general, the main goal is to engage patients more actively in their health and care.

Lavallee et al. [7] describe several primary sources of PGHD that are being used in healthcare settings. Patients are generating PGHD through the use of wearable devices and mobile health apps that allow them to track metrics such as physical activity, heart rate, blood pressure, weight, sleep, and more. This type of data is often used by patients for their own health monitoring and management, or is shared with healthcare providers to facilitate data-driven care. Other sources of PGHD include patient portals and electronic health records, which allow patients to directly submit information like food/symptom logs, mood ratings, and customized health data to their providers. Validated clinical assessments and symptom trackers are also sources of PGHD that allow systematic tracking of patient-reported outcomes over time. Patients and caregivers are also using journals and diaries to capture highly customized PGHD that may not be easily recorded through devices and apps. On the provider side, healthcare systems are using PGHD for remote patient monitoring programs, particularly for chronic disease management. Telehealth technologies allow PGHD to expand care access and oversight. Finally, both patients and providers described participating in clinical research efforts to collect PGHD, often with the goal of informing future clinical use cases and system-wide implementation. In summary, PGHD is generated from a diverse set of sources, including wearables, apps, portals, assessments, diaries, and research initiatives. Patients and providers are leveraging these sources of PGHD to enable data-driven care, patient engagement, population health, and care access improvements. However, fully integrating PGHD into clinical workflows and health IT systems remains an ongoing challenge.

Nittas et al. [67] state that PGHD originates primarily from health care consumers themselves, transforming their daily lives into a valuable source of health information. In healthcare settings, PGHD is increasingly used for a variety of purposes. The article highlights that a majority of studies integrate electronic PGHD with elements such as reflection, guidance, motivation, and education, which form a core part of digital prevention strategies. In particular, many of these interventions are fully automatized, demonstrating a shift towards resource-efficient care. These data not only strengthen proactive and person-centered healthcare approaches, but also play a pivotal role in disease prevention, health promotion, personalized counseling, and remote monitoring. The increasing reliance on PGHD underscores the evolving landscape of healthcare care, emphasizing digitalization and a more active role for consumers in their health management.

Austin et al. [95] examined various case studies of the use of PGHD in a healthcare system. They found that wearable devices and mobile/sensor technologies were the most common platforms for PGHD collection, used in 41% and 47% of case studies, respectively. These devices were used to collect data on physical activity, sleep, location, mood symptoms, and other metrics that reflect daily health and behaviors. The key goals cited for the use of PGHD were to improve the monitoring between clinical visits (8 case studies), personalize care plans (4 case studies) and assess outcomes (3 case studies). By capturing data on patient experiences and health patterns outside of clinical encounters, PGHD aimed to provide a more comprehensive view of health status to support care decisions. Overall, the use of wearables and mobile apps to collect patient-generated data allowed more continuous, personalized monitoring and care delivery in healthcare settings.

According to Bourke et al. [59] PGHD from sources such as smartphones, wearables, social media, and patient registries is emerging as a valuable complement to traditional healthcare data. Patients can actively contribute by recording symptoms, medication use, patient-reported outcomes on platforms such as apps and patient forums. Passive data collection via sensors in devices like smartwatches and ingestible pills also provides insights not typically captured in medical records, such as continuous physiologic parameters, detailed behaviors, and environmental exposures. The unique value of PGHD is that it offers the patient’s perspective-experiences, attitudes, lifestyle factors, treatment adherence patterns, and more. While new methodological considerations exist, PGHD is being used to enable patient-centered research and care, capture data missing from routine clinical sources, study factors influencing health behaviors and outcomes, validate other data sources, and ultimately allow a more complete understanding of therapeutic interventions. Careful incorporation of this new patient-contributed data stream offers innovative opportunities for pharmacoepidemiology and improving health services.

Table 4 presents the insights and findings from the above discussion on primary source and utilization of PGHD in Healthcare Settings in a tabulated format.

### 5.2. RQE2: How Do Patients Perceive the Collection and Utilization of Their Generated PGHD?

Zhu et al. [68] in their interview study found that patients are motivated to collect PGHD to gain self-awareness and self-management skills, also tracking health data gives them insight into how their lifestyle and behaviors affect their health. They want to collect comprehensive PGHD to share with clinicians and collaborate on their healthcare and see it as a way of having informed discussions with clinicians. Some patients collect PGHD out of curiosity and seeing the effectiveness of treatments and collecting the data gives them hope and helps them feel that they are actively participating in their care. Patients want to use PGHD to alter treatment plans or medications also. They collect data to provide justification for changing medications or stopping medications. Sharing PGHD during clinical visits is perceived by some patients as burdensome. They have to select what is most relevant to share, given a limited visit time. Some patients perceive that their clinicians are not receptive to the PGHD they collect and initiate sharing on their own. This discourages them from collecting and sharing the data. Patients want clinicians to provide guidance on what PGHD is clinically relevant to collect. This would make the data more useful for clinicians to use. Patients want to be equal partners and collaborate with clinicians on their health care. Sharing PGHD is seen as a way to engage clinicians and alter treatment plans. In summary, patients are motivated to collect comprehensive PGHD to gain personal insight, share with clinicians, and collaborate on their care. But they need the guidance of clinicians on what is clinically relevant to collect. They perceive time constraints and clinician receptiveness as barriers to fully utilizing PGHD.

Lavallee et al. [7] interviews revealed that patients are intentional about when and what type of PGHD they share with providers. PGHD tracked solely for personal health goals or wellness purposes was typically not shared in formal healthcare settings. Patients decided to share PGHD with providers when they felt that it could support care decisions, improve communication about their health status and concerns, or provide data to inform treatment choices. Patients recognized that appointment time is limited, so sharing targeted PGHD is one way to maximize discussions and prepare in advance. However, if patients perceived that providers did not find the PGHD useful or relevant, they would be less likely to continue sharing it. Some patients even sought new providers who were more receptive to integrating PGHD into care. In general, patients were selective about sharing PGHD and considered factors such as perceived provider interest, relevance to care decisions, and visit time constraints when determining what data to bring to healthcare encounters.

Burns et al. [69] in an interview with the patient found that they perceive the collection and utilization of their generated PGHD as an overwhelmingly positive experience. Generating and using their own visual health data allows patients to feel more engaged, empowered, reassured, and in control when it comes to managing their health situations. The ability to visually track progress over time provides confidence that their conditions are improving. It aids comprehension and memory-making around their health journeys. Patients feel a greater sense of responsibility and control over their health outcomes by actively participating in monitoring and documentation. PGHD improves doctor-patient communication by providing visual evidence to accompany verbal descriptions of health problems. In general, patients find that collecting and using PGHD engages them more actively in their own care, improves doctor-patient relationships, and allows them to feel more empowered and confident through self-monitoring their conditions.

Adler-Milstei et al. [3] discuss that the patients’ perceptions of the collection and utilization of their PGHD are multifaceted. They generally find that the types of data collected—health history, validated questionnaires and surveys, and biometric and health activity—align with their preferences and needs, with the notable exception of biometric and activity data collected by third parties, which raises privacy concerns. Patients are motivated to work with PGHD to achieve specific health goals, improve their understanding of their health, make office visits more efficient, and assist their providers in diagnosis and care management. They have reported positive results when PGHD was used effectively, such as achieving accurate diagnoses after previous errors. However, there are challenges in maintaining patient engagement with PGHD, including the manual effort and technical know-how required for data submission and the lack of clear communication from providers about the value and use of data. Patients do not expect immediate feedback on the PGHD they provide, but have noted problems with how these data are integrated into their interactions with healthcare providers. They also stress the importance of differentiating PGHD from data generated by clinicians within electronic health records. Furthermore, the communication between patients and providers is critical; patients are more likely to submit data if they understand its value to their care and if providers demonstrate how, it informs clinical decisions. Policy-related challenges also emerge, particularly regarding the lack of reimbursement for PGHD and potential liability issues, which hinder the integration of PGHD into clinical workflows. Overall, while patients recognize the potential benefits of PGHD, their concerns about privacy, the submission process, and the clarity of its use in their healthcare are significant and need to be addressed to improve engagement and integration.

Kim et al. [70] found that patients have mixed perceptions regarding the collection and utilization of Patient-Generated Health Data (PGHD). On the one hand, they acknowledge that PGHD can improve transparency in the patient-provider relationship, potentially leading to better care. However, this transparency also raises concerns, as some patients may respond negatively by stopping collecting data, selectively sharing information or manipulating the data to appear compliant, driven by the desire to maintain a positive relationship with their healthcare providers. Clinicians recognize the value of PGHD despite possible inaccuracies and express concerns about the reliability of the data due to the reporting habits of patients. Older adults, in particular, find the monitoring aspect of PGHD intrusive and threatening their autonomy, leading to resistance. The need for education on how to collect and use PGHD is evident, but the responsibility to provide this education remains unclear, especially when the monitoring is initiated by patients themselves. There is also a noted uncertainty about how clinicians interpret PGHD and use it to benefit patient health. There is a mismatch between the available technology and the actual needs of patients and clinicians, with a preference for passively collected data due to its perceived accuracy. In general, while PGHD is seen as a tool that could potentially improve clinical care, it also presents challenges related to trust, privacy, and the balance between its benefits and its perceived intrusiveness.

Smith et al. [71] explore the perspectives of cancer survivors about providing patient-generated health data and patient-reported outcomes to central cancer registries. His study highlights significant insights into the perspectives of patients on the handling of their patient-generated health data (PGHD). Initially unfamiliar with the concept of cancer registry, patients became comfortable contributing diverse types of PGHD once they understood the objectives of the registry. Their motivation to share hinged on the altruistic potential to help fellow patients. Although some assumed that their information would remain private, others stipulated confidentiality as a prerequisite for sharing. They were prepared to provide data covering medical history, symptomatology, quality of life, functional ability, care experiences, lifestyle and economic factors. In particular, there was a prevalent demand for the inclusion of data on the enduring effects of cancer and the repercussions of treatments. Patients not only sought their data to enrich treatment options and facilitate their adaptation process but also to gain access to reciprocal information from the registries. This information ranged from side effects and survival rates to novel treatments and insights into quality of care. The preferred methods for submitting PGHD varied, including mail, phone, online surveys, and integration with patient portals. In essence, there is a strong willingness among patients to share their PGHD with registries in the hope of benefiting others, coupled with a clear expectation of privacy and the desire to receive valuable information in return.

Table 5 presents the insights and findings from the above discussion on patient perceptions and utilization of PGHD in a tabulated format.

### 5.3. RQI1: What Are the Potential Ramifications of Data Breaches Pertaining to PGHD on Trust and Healthcare Outcomes?

Ostherr et al. [72] highlight key points emerge with respect to patient trust, data breaches, and health outcomes: Despite the high-profile data breaches, patients expressed little concern about sharing their health data with corporations. Some felt that the transactional nature of consenting to the terms of service overrode privacy concerns. However, when approached for research purposes and informed about the risks of data privacy, the patients became more wary. This suggests an asymmetry in which companies face less scrutiny than researchers for the handling of health data. Many patients felt that sharing data in general could improve health outcomes more than lab research alone. However, recruiting participants was still difficult when privacy risks were highlighted. There is a disconnect between attitudes on corporate data sharing versus research/clinical data sharing. The public discourse on data privacy threats also contrasts with lax reading of terms of service.

The regulatory environment lags behind real-world data sharing practices and attitudes. More public dialogue on the benefits/harms of health data is needed. In summary, the conclusion focuses on how patient trust differs based on context, the need to update regulations, and the complex relationship between data sharing and perceived health outcomes. Although patients tolerate corporate data practices, highlighting risks reduces trust, signaling a need for greater transparency.

However, some potential ramifications of data breaches of PGHD on patient trust and healthcare outcomes include the following:

- Loss of trust in companies and technologies collecting patient health data. The article mentions users’ comfort in sharing health data with corporations, despite the potential for exploitation. Data breaches could erode this trust and make patients more cautious about using health apps and devices.
- Unwillingness to share health data. The article notes that researchers already face challenges in getting people to share health data for studies due to privacy concerns. Data breaches could exacerbate this and inhibit research using patient-generated data.
- Negative impacts on care. The article suggests that patient-generated data could improve outcomes by providing information on health/illness. If data breaches discourage patients from sharing data, it could hinder these potential health benefits.
- Reluctance to disclose sensitive information. Patients may be less forthcoming with potentially relevant health details during clinical encounters if concerned about data privacy and breaches. This could negatively impact care.
- Increased stress/anxiety. Data breaches involving sensitive health information could lead to emotional distress for patients whose data is exposed. This psychological impact could also take a toll on wellbeing.

In summary, by undermining patient trust and inhibiting data sharing, health data breaches could limit the progress and benefits expected from access to patient-generated data, leading to detrimental impacts on care, health research, and patient well-being. Maintaining rigorous data privacy and security is critical to realizing the potential of PGHD.

### 5.4. RQI2: What Impact Does the Incorporation of Patients and Their PGHD Have on Healthcare Decision-Making?

According to Jim et al. [73] PGHD can have several important impacts on healthcare decision making:

- Improving symptom monitoring and management: PGHD, such as patient-reported outcomes (PROs), allow for more comprehensive and real-time assessment of patient symptoms and side effects. This enables earlier detection and treatment of issues that can impact quality of life or clinical outcomes.
- Informing treatment choices: PGHD provides additional information on patient experiences that can better inform shared decision-making about treatment options. For example, PRO data on side effect profiles can help patients and physicians select the optimal treatment regimen.
- Predicting health events: The longitudinal analysis of PGHD can uncover patterns predictive of disease progression, treatment complications, or health emergencies. This can allow for preventive interventions. For example, a rapid decline in activity monitoring data could indicate an upcoming need for hospitalization.
- Population health management: PGHD aggregates can help identify trends, disparities, and opportunities to improve care delivery across patient populations. This is useful for quality improvement initiatives.
- Clinical trial outcomes: Incorporating PGHD as secondary endpoints in the trials provides a more comprehensive view of treatment effects on quality of life and symptomatic adverse events from the perspective of the patient.
- Regulatory decisions: Drug and device approvals are increasingly informed by patient experience data from PGHD. This ensures that the patient’s voice is represented in the benefit-risk determinations.
- Remote patient monitoring: PGHD enables care outside traditional settings through telehealth, reducing hospital visits. During the COVID-19 pandemic, remote monitoring using PGHD became especially important.

In summary, PGHD allows care to be tailored to patients’ unique needs and preferences, predict risks, and improve health outcomes across entire populations. Its integration into decision-making processes can improve patient-centeredness, quality of care, and value.

Singh et al. [74] elaborate the impact of incorporating PGHD on healthcare decision-making are:

- PGHD from mobile apps and wearables provides additional and more up-to-date information about the patient compared to only relying on healthcare professional-generated data (HPGD) from EHRs. This can lead to faster and improved healthcare decision making.
- PGHD allows continuous monitoring of patient health parameters such as adherence to medications, exercise, sleep, etc. These real-time data can enable timely interventions and personalized care that lead to better health outcomes.
- Integration of PGHD and HPGD provides a more holistic view of the patient’s health. Decisions can be based on both historical clinical data as well as current lifestyle and health indicators tracked by the patient.
- Patient participation and empowerment increase with PGHD as they take greater ownership over monitoring and managing their health. This can improve their adherence to care plans.
- Quality of decision-making can potentially be improved by speeding up decision time, reducing errors, improving patient outcomes, and reducing healthcare costs. Metrics to quantify improvements need further research.
- Challenges like data integration, privacy/security, regulatory issues, impact on provider workload need to be addressed for effective utilization of PGHD in decision making.

In summary, incorporating patient-generated data facilitates patient-centered care by engaging patients in their own health management. This, together with the integration with clinical data, provides a foundation for more informed and timely shared decision-making between providers and patients.

Petersen et al. [35] discuss the significant role of PGHD in the medical decision-making process. It highlights that patients are actively involved in collecting and sharing their health data in order to improve their treatment outcomes. Such proactive sharing facilitates more productive and informed consultations with healthcare providers. Furthermore, PGHD contributes to greater patient participation and equips physicians with valuable information about the health of their patients beyond the clinical environment, thereby improving the decision-making process. When PGHD is reviewed collectively, it not only promotes better health results, but also encourages patients to participate more in activities beneficial to their health. Transparent exchange of PGHD also encourages honesty in patient-provider interactions, leading to decisions grounded in accurate information. Despite these benefits, the lack of standardized protocols for PGHD is a significant obstacle to its full integration into clinical practice. In conclusion, the inclusion of PGHD enriches the clinician-patient dynamic, improves collaborative decisions, and promotes improved health outcomes, although standardization and effective integration into healthcare systems remain areas for development.

Cohen et al. [6] examine the impact of incorporating PGHD in clinical decision-making and care. The authors interviewed clinicians and researchers involved in five studies testing PGHD collection tools. They found that PGHD provides valuable insights between visits and reveals patient problems that may be missed during appointments. Incorporating PGHD into healthcare care provides clinicians with a comprehensive view of a patient’s health over time, allowing for better disease management and more personalized care. PGHD offers detailed information that may be missed in routine visits, helping to adjust care plans more effectively and potentially reducing unnecessary clinic visits. However, it requires the development of specific protocols for data collection, integration into EHR and response to data, while also considering the privacy and security of the stored information. Moreover, PGHD empowers patients by giving them control over their health data, fostering greater autonomy, and transforming the patient-clinician dynamic.

Wood et al. [75] explains that the incorporation of PGHD into healthcare decision making can improve treatment efficacy, identify patients at risk of treatment toxicity, and inform personalized treatment plans. PGHD allows for a better understanding of the long-term impacts of treatments on quality of life, informs the conduct of clinical trials, and provides prognostic value in clinical outcomes. In addition, PGHD can contribute to the evaluation of new therapeutics and potentially influence regulatory decisions. By tracking metrics such as symptoms, medication adherence, and lifestyle factors, PGHD can also play a role in clinical research to improve patient well-being. In general, PGHD promotes more personalized and informed healthcare decisions that benefit patient experiences and outcomes, while also advancing clinical research.

Petersen et al. [76] discusses policy recommendations to facilitate the use of PGHD from apps, wearables, etc. in shared decision making between patients and clinicians. Incorporating PGHD makes care more patient-centric by bringing in the patient perspective. Accessing their own data also allows patients to take an active role in managing their health. For clinicians, PGHD provides a more holistic view of patients for tailored care plans and allows tracking of health status over time. At the population level, aggregated PGHD can reveal health trends to guide public health efforts. However, concerns such as privacy risks, ensuring accuracy, building clinician trust in PGHD, and avoiding marginalization need to be addressed. The authors suggest policy changes such as stronger data protections and consent processes so patients can trust that their data are safe. Patients should be able to easily access their PGHD. The collection and use of PGHD should be transparent and ethical. While PGHD holds promise in healthcare decision-making, policies must balance maximizing its benefits with minimizing potential harms, especially for vulnerable groups. If implemented effectively, PGHD can make care more collaborative and empower the patient.

Table 6 presents the insights and findings from the above discussion on impact on incorporation of patients and their PGHD in healthcare decision-making in a tabulated format.

### 5.5. RQS1: What Are the Recommended Strategies for Improving the Security and Privacy of PGHD?

Improving the security and privacy of PGHD involves several strategies:

(a) Restrict Access to Data and Applications: Limiting who can access PGHD can help prevent unauthorized use. To ensure the security and privacy of patient information inside healthcare systems, many main tactics are employed. These include the implementation of multifactor authentication (MFA) [77], the use of role-based access control (RBAC) [78], the adherence to the principle of least privilege, conducting frequent audits, and the deployment of firewalls and virtual private networks (VPNs) [79]. Multifactor authentication (MFA) improves security by necessitating the provision of two or more distinct forms of proof by users as a prerequisite for access, such as a password and a fingerprint. Role-Based Access Control (RBAC) is a security mechanism that allocates user roles and permissions according to the specific requirements of their job responsibilities. This approach guarantees that users are granted access only to the data that are essential for the execution of their designated duties. The idea of least privilege enhances control measures by restricting user access to only the essential level required to perform their responsibilities. Implementing routine audits plays a critical role in the identification of security vulnerabilities and the enforcement of approved access protocols. Firewalls serve as protective measures against illegal network invasions, whilst Virtual Private Networks (VPNs) provide the security of remote access. In conclusion, maintaining regular software updates, encompassing both security and medical applications, is vital to protect against any vulnerabilities that might be exploited. These techniques together strive to ensure the security of patient data, allowing access exclusively to individuals who have been authorized for legal purposes.
(b) Implement Data Usage Controls: The implementation of data usage controls in the healthcare sector is of utmost importance, also in order to protect PGHD and ensure that its utilization is in accordance with patient permission and legal obligations. This involves the implementation of robust access controls to restrict data visibility only to authorized individuals, the use of encryption techniques to safeguard data integrity, and the establishment of audit trails to ensure accountability. Strict adherence to regulatory frameworks, such as the Health Insurance Portability and Accountability Act (HIPAA) in the United States and the General Data Protection Regulation (GDPR) in Europe, is imperative and leaves little room for negotiation. Consequently, it is essential to establish and maintain robust systems to ensure compliance with these requirements. It is imperative for healthcare companies to establish and maintain explicit policies and procedures that undergo continuous updates in order to align with the ever-changing legal and ethical norms. It is important to provide training to healthcare personnel about data privacy and security protocols, while also ensuring that patients are adequately informed about their rights and the extent of their control over their personal data. The involvement of stakeholders in the process of policy creation and the establishment of effective communication channels are crucial in ensuring that regulations on data usage are well-informed and comprehensive. Continuous monitoring, frequent audits, and feedback loops play a crucial role in facilitating continual progress, while being abreast of technology changes can further bolster endeavors aimed at safeguarding data. Collectively, these steps establish a safe milieu for the management of PGHD, cultivating confidence among individuals and guaranteeing the responsible and efficient utilization of their data for healthcare provision.
  Petersen et al. [76] underscores the importance of enhancing the security and privacy of PGHD by recommending several key strategies. Policymakers are called upon to fortify privacy and security protections for PGHD, bridging gaps in existing laws and ensuring strict enforcement. It advocates for individual access to PGHD in real-time and electronically, reinforcing transparency and personal data control. The need to clarify informed consent processes is emphasized, ensuring individuals are well-informed about how their data is managed and shared, with the flexibility to revise their consent. The use of PGHD should be ethically bound, with a prohibition on potentially harmful non-health applications. Additionally, the article suggests individuals should have the option to securely share their PGHD for research purposes, with privacy intact. Regular review processes for PGHD analytics, updated HIPAA regulations to reflect current practices, and clear, enforceable penalties for data misuse are also recommended. Collectively, these measures aim to establish robust policy changes, augmenting privacy, security, and ethical use of PGHD while giving individuals enhanced control over their data.
(c) Logs and Monitor Use: Keeping track of who is accessing PGHD and when can help identify any potential security breaches. Prosper et al. [80] emphasize the increases in healthcare data breaches and highlight the need for analyzing security practices like access logs in EHR systems which could also relate to PGHD. The paper simulates EHR logs data since real logs are highly confidential. The simulated logs have normal accesses and some anomalous accesses imitating attackers. For role classification, Decision Tree and Random Forest perform best with 0.89 accuracy. For anomaly detection, all methods have high recall but low precision. Soft classification works better than hard classification. Bernoulli Naive Bayes on non-normalized data performs best with F1-score of 0.893 for anomaly detection. The high recall means the methods can help narrow down data for further investigation of anomalies by hospital IT staff. Future work is needed to better distinguish between anomalies and true malicious events. More comparisons on real data could also help. The paper proposes an anomaly detection method to detect potentially illegitimate access to patient records in electronic health records (EHR) systems. The method uses machine learning algorithms to classify user activities into roles. It then detects anomalies by comparing a user’s daily activity to the expected activity for their role. Several machine learning algorithms are evaluated including Naive Bayes, SVM, Neural Networks, KNN, Logistic Regression, Random Forest, and Decision Trees.
(d) Encrypt Data at Rest and in Transit: Encryption can protect PGHD from being intercepted or accessed by unauthorized individuals. Shaik et al. [81] presents a novel cryptographic algorithm designed to secure unencrypted non-data database files both at rest and in transit. Recognizing the vulnerability of non-data files, which are often overlooked by standard encryption methods like Transparent Data Encryption (TDE), the authors propose a two-level encryption logic that incorporates a passcode lock, ensuring that file owners maintain complete control over their encryption. This approach addresses the security and compliance shortcomings of third-party encryption tools by providing a robust, in-house solution for protecting sensitive database information from unauthorized access. The practical application of this algorithm is validated with real-world results, showcasing its effectiveness in enhancing the security of database files beyond the capabilities of existing methods.
(e) Secure Mobile Devices: Many healthcare providers use mobile devices in their work, and also the patient uses the mobile devices to collect PGHD these can be a potential security risk if not properly secured. Vrhovec et al. [82] examines the proliferation of mobile devices in healthcare and the consequent security risks, noting that 44% of data breaches in healthcare are linked to mobile device use. It highlights the benefits of mobile devices, such as improved healthcare worker coordination and reduced data redundancy, while also addressing the significant security challenges posed by rapid technology adoption, including device theft, insecure applications, and inadequate security management. To mitigate these risks, the paper recommends establishing comprehensive security management practices, including administrative measures, physical security, secure data exchange, continuous adaptation to technological changes, and particularly emphasizes the importance of training and raising awareness among healthcare workers about the secure use of mobile devices.
(f) Mitigate Connected Device Risks: Devices that are connected to the internet can be vulnerable to hacking, so steps should be taken to mitigate these risks. To mitigate the risks associated with connected medical devices Yaqoob et al. [83] explain a multifaceted approach is essential. This includes understanding and addressing programming issues in legacy devices, adhering to FDA regulations by reviewing pre-market submissions for safety and security, and implementing robust access control mechanisms. Additionally, the development of lightweight secure algorithms is crucial for resource-constrained implantable and wearable devices to prevent attacks that compromise confidentiality, integrity, and availability. Attestation-based architectures are also recommended to protect against run-time attacks that exploit programming vulnerabilities. Furthermore, ongoing research and development are imperative to analyze and improve upon current countermeasures, ensuring the security of these critical devices in the face of sophisticated cyber threats. Communication technologies and standards must be scrutinized for security features, with a focus on both wireless and wired technologies used by these medical devices.
(g) Conduct Regular Risk Assessments: Regular assessments can help identify any potential weaknesses or vulnerabilities in the system. Yaqoob et al. [84] implementation of the Integrated Security, Safety, and Privacy (ISSP) Risk Assessment Framework for medical devices involves a systematic approach that includes identifying security vulnerabilities and safety-related bugs, considering past issues, and predicting future ones. The framework uses FDA recalls considering past software/electrical/mechanical (SW/EM/UI) issues and predicts their impact on patient safety. It also incorporates the Common Vulnerability Scoring System (CVSS) to assess security vulnerabilities and FDA recalls evaluating SW/EM/UI failures. The framework calculates the ISSP risk of medical devices by considering the unified impact of safety, security, and privacy risks on patient well-being. It suggests appropriate security controls with respect to the device’s classification, addressing vulnerabilities that could affect patient privacy and health. The framework is compared against NIST best practices, ISO standards, and FDA guidance documents, demonstrating its systematic approach to determining security, privacy, and safety risks and suggesting security controls relative to the device’s class. For further details on the implementation steps, the document outlines the important steps of the ISSP framework, which are exhibited in a figure within the document and discussed in subsequent sections. This includes the identification of events that trigger hazards and threats, analysis of available controls required by regulatory bodies for device approval, and the emphasis on the criticality and sensitivity of data and information. The framework is validated using a case scenario of an infusion pump and is evaluated by comparing it to current practices, showing that it provides a unified approach to considering different types of risks associated with medical devices.
(h) Educate Healthcare Staff: The human element remains one of the biggest threats to security across all industries, this is also case in the healthcare field. Nifakos et al. [85] outlines the importance of educating healthcare staff to reduce security threats through a combination of strategies. It emphasizes the need for collaborative and standardized training programs and awareness campaigns that inform on the nature and types of cyber threats. Emphasis is placed on maintaining cyber hygiene and information governance through mandatory training, which is crucial for understanding risks, particularly regarding information leakage on social media. The document also highlights the effectiveness of tailored training programs and simulation exercises for management executives to underscore the often-overlooked importance of cybersecurity compared to other emergencies. Best practices recommended by healthcare experts should be widely promoted among all healthcare stakeholders, and there is a call for the development of formal training and educational standards to address human factors in cybersecurity. Additionally, it is suggested that IT systems should be adept at detecting social engineering attacks, while healthcare professionals should be equipped with the knowledge to recognize such threats. The document concludes that there is a critical need for a systematic methodology to harmonize research findings for objective evaluation by cybersecurity experts in the healthcare industry.

In addition to these, it’s important to consider the integration of PGHD into electronic health records (EHRs).This process is still in its early stages, and efforts are needed to understand how to optimize PGHD integration into EHRs considering resources, standards for EHR delivery, and clinical workflows.It’s also crucial to develop a secure architecture compatible with the EHR vendor proprietary non-FHIR web services.

### 5.6. RQS2: In What Ways May Healthcare Providers and Technology Developers Engage in Collaborative Efforts to Achieve an Ideal Equilibrium between the Value of PGHD and the Safeguarding of Data Privacy and Security?

The research suggests various ways in which healthcare providers and technology developers can collaborate to balance the value of PGHD with data privacy and security:

(a) Integration of PGHD into Clinical Care: PGHD collected through technical applications can provide deeper insight into a patient’s condition and health between clinic visits, which can lead to more accurate patient information and revised care plans for improved health goal achievement. This can also reduce unnecessary clinic visits [6].
(b) Overcoming Barriers: While wearable devices and mobile health apps allow individuals to collect PGHD outside of healthcare encounters, which can improve patient-provider communication and engagement, significant barriers include data validity, actionability, and the burden of integrating PGHD into existing care processes [7].
(c) Data Sharing and Anonymity: Healthcare organizations can collaborate to disclose significant quantities of personal biomedical data without violating anonymity, which suggests that data privacy can be maintained while sharing valuable health data [86].
(d) Privacy and Public Benefits: Analysis of real-world data can offer stakeholders practical protocols/guidelines for publicizing patient information and design implications for future systems, such as automatic privacy sensitivity checking, to strike a balance between privacy and public benefits [87].
(e) Patient Concerns and Health System Challenges: Health systems face challenges related to lack of reimbursement, data quality, and clinical usefulness of PGHD. Patients have concerns about data security and the value of reporting, which must be addressed [3].
(f) Governance Framework: To strengthen the social license for data sharing, conditions such as value, privacy, risk minimization, data security, transparency, control, information, trust, responsibility, and accountability must be operationalized in a governance framework [88].
(g) Routine Data Sharing: Doctors routinely share health data electronically under HIPAA, and sharing with patients and patients’ third-party health apps should be just as routine, provided it is consistent with privacy regulations [89].
(h) Privacy-Preserving Data Aggregation: A privacy-preserving health data aggregation scheme can securely collect health data from multiple sources and guarantee fair incentives for contributing patients [90].
(i) Ethics and Privacy Framework: An integrated approach to ethics and privacy can help achieve consensus on privacy and ethics principles, which could accelerate health data access in data-driven research projects [91].
(j) Research Directions for Privacy Concerns: There is a need for research directions for techniques and mechanisms to address patient’s data privacy concerns in cloud-assisted healthcare systems [92].
(k) EHR Integration Efforts: Mobile health app developers should examine PGHD information needs to inform Electronic Health Record (EHR) integration efforts [93].
(l) Policy Considerations for Interoperability: Policy considerations for improving specific aspects of health information’s interoperability while preserving patient data privacy and security are necessary [94].

These findings indicate that a multi-faceted approach involving technical, ethical, legal, and policy considerations is essential for the successful integration of PGHD while ensuring data privacy and security.

### 5.7. RQF1: How Can PGHD Prepare for the Upcoming Security and Privacy Problems Brought on by the Widespread Adoption of AI and ML in Healthcare?

Below the Table 7 includes more detailed descriptions focusing specifically on PGHD and security measures for AI and ML applications in healthcare:

These detailed measures provide a framework for securing PGHD in the context of AI and ML in healthcare, ensuring that as technology advances, patient privacy and data integrity remain at the forefront of healthcare initiatives.

Also, we include more of those advanced security measures in the context of securing PGHD for AI and ML applications in healthcare in the Table 8 below:

### 5.8. RQF2:How May the Next Decade’s Development of Wearable Technology and Internet of Medical Things Devices Affect the Current State of PGHD Security and Privacy Concerns?

The development of wearable technology and Internet of Medical Things (IoMT) devices over the next decade is likely to have a significant impact on PGHD security and privacy concerns in several ways:

(a) Increased Data Volume [26]: As wearable technology becomes more prevalent, the volume of PGHD will increase exponentially. This will pose greater challenges in securing the data against unauthorized access and ensuring privacy, as there will be more data points and potentially more sensitive information available.
(b) Advanced Data Analytics [21,37]: With more data, there will be a push for advanced analytics to make sense of the information. This could lead to the development of more sophisticated algorithms for data processing, which could either strengthen data security (by identifying and mitigating breaches more quickly) or create new vulnerabilities (if the algorithms themselves are not secure).
(c) Improved Security Measures [77,78,79,81]: The rise in security threats could drive innovation in security technologies. We might see the development of more robust encryption methods, secure data transmission protocols, and advanced user authentication processes specifically designed for wearable and IoMT devices.
(d) Regulatory Evolution [23]: As technology advances, regulations and standards will need to keep pace. This could mean new or updated legislation around data protection specific to health data generated by wearables and IoMT devices, which could help address privacy concerns.
(e) Public Awareness and Education [85]: With the proliferation of these devices, consumers may become more aware of the potential risks to their data. This could lead to increased public demand for better security and privacy protections, which in turn could pressure manufacturers and service providers to prioritize these concerns.
(f) Integration Challenges [24,25,27,53,58]: As wearables and IoMT devices become more integrated with other healthcare systems, the complexity of ensuring end-to-end security and privacy protections increases. There will be a need for standardized protocols and interoperability frameworks that can maintain security and privacy across different platforms and devices.
(g) Ethical and Legal Implications [6,23]: The next decade may also bring to the forefront ethical questions about data ownership, consent for data use, and the balance between individual privacy and public health benefits. This could reshape the landscape of PGHD security and privacy concerns, leading to new legal precedents and ethical guidelines.
(h) Market-Driven Solutions [95]: As competition in the wearable technology and IoMT space intensifies, companies may differentiate themselves through superior privacy and security features. This could lead to market-driven improvements in PGHD security and privacy.
(i) Personalization of Security [112]: There may be a trend towards personalized security settings, where users can set their own preferences for data sharing and privacy, giving them more control over their PGHD.
(j) Global Disparities [73]: The impact on PGHD security and privacy concerns will likely be uneven across the globe, with disparities in technological advancement, regulatory environments, and public awareness affecting how these issues are addressed.

It’s important to note that these are projections, and the actual impact will depend on a variety of factors, including technological breakthroughs, market dynamics, regulatory changes, and shifts in consumer behavior.

## 6. Discussion

### Research Questions Discussion

RQE1 [7,27,34,59,66,67,95] helps to identify the major sources of PGHD include mobile health applications, wearable devices, patient portals and personal health records, remote monitoring sensors/IOM devices, virtual/mixed reality systems, social media platforms, validated assessments, and patient diaries. Patients are using these technologies to collect, track and share various health metrics with providers, spanning symptoms, lifestyle behaviors, physiological data, and customized patient-reported outcomes. Healthcare systems are increasingly using PGHD to enable patient self-monitoring/management, facilitate continuous care between visits, capture patient perspectives to personalize treatment plans, support clinical decision making with more comprehensive data, assessing outcomes, informing system design, promoting patient engagement, and advance clinical research including treatment effectiveness and precision medicine efforts. While PGHD integration offers innovations in research and care delivery, open questions remain around ensuring data quality, system interoperability, and appropriate use regulations.

RQE2 [3,7,68,69,70,71] focuses on exploring how patients view the collection and use of their PGHD. Insights from interviews conducted by various researchers reveal that patient perspectives encompass multiple aspects, which include:

- Motivation and Barriers: Patients collect PGHD to improve self-awareness, manage their health, assess treatment effectiveness, provide reasons for changes in medications, and collaborate with healthcare professionals. However, they face challenges in discerning clinically relevant data and dealing with obstacles such as short appointment durations and varying levels of clinician engagement.
- Selective Sharing: When deciding to share PGHD, patients weigh factors such as the healthcare provider’s interest, the relevance to care, and time constraints during appointments. If they perceive a lack of receptiveness from providers, patients may withhold information or seek alternative healthcare professionals.
- Impact on Patient Engagement: Using PGHD can make patients feel more involved, empowered and reassured about their health management. It also improves their understanding, memory, communication with physicians, and responsibility for health outcomes.
- Perceptions and Concerns: Patients acknowledge the benefits of PGHD but also express significant worries about privacy, the process of data submission, understanding of PGHD use, and the trade-off between benefits and intrusiveness. Addressing these concerns is crucial for enhancing patient engagement.
- Familiarity and Willingness to Share: Initially, patients may be unfamiliar with the uses of PGHD but tend to become more open to sharing it once they comprehend its objectives, especially when motivated by the potential to aid others. Confidentiality is a key concern, and patients often expect reciprocal access to information.

In conclusion, while patients recognize the potential advantages of PGHD, they also identify significant challenges related to the process, privacy, communication, and mutual benefits. These issues must be resolved to foster more effective and sustained patient engagement and use of PGHD.

RQI2 [6,35,73,74,75,76] emphasize the incorporation of PGHD and its understanding from sources like mobile apps and wearables has wide-ranging impacts on healthcare decision-making. PGHD provides more comprehensive, real-time data about patients’ health and experiences, enabling more informed, personalized, and timely treatment decisions. It also facilitates continuous remote monitoring, allowing earlier interventions for better outcomes. While promising, utilizing PGHD poses some challenges regarding privacy, accuracy, integration into clinical workflows, and avoiding marginalization of disadvantaged groups. However, overall PGHD is transforming decision-making to be more patient-centered and collaborative. Through participatory tracking and data sharing, patients are empowered in managing their health. Meanwhile clinicians gain fuller understanding of patients for tailored care plans. Effective policies must balance maximizing PGHD’s benefits for decision quality while minimizing potential harms.

RQS1 [76,77,78,79,80,81,82,83,84,85] explores several important strategies that can help safeguard the security and privacy of PGHD. These include restricting data access to authorized personnel only, implementing robust data usage controls, maintaining audit trails to monitor data access, encrypting data at rest and in transit, securing mobile devices used to collect PGHD, mitigating risks associated with connected devices, conducting regular risk assessments to identify vulnerabilities, educating healthcare staff about security protocols, and carefully considering how to integrate PGHD into electronic medical record systems. Employing a multilayered approach that encompasses technological solutions, strong policies, and human accountability can help provide comprehensive protection for sensitive patient information.

RQS2 [3,6,7,86,87,88,89,90,91,92,93,94] investigates how healthcare providers and technology developers can collaborate effectively to balance the value of PGHD with the safeguarding of data privacy and security. The research identifies multiple approaches for this: integrating PGHD into clinical care for more accurate patient information and improved health goals; addressing barriers like data validity and integration challenges; maintaining data privacy while sharing valuable health data; developing practical protocols for balancing privacy with public benefits; addressing patient concerns and health system challenges regarding data security and the usefulness of PGHD; creating a governance framework that operationalizes value, privacy, and risk minimization; making routine data sharing with patients and their apps as common as current health data exchanges; implementing privacy-preserving data aggregation schemes; adopting an integrated approach to ethics and privacy in health data; researching techniques to address privacy concerns in cloud-assisted healthcare; focusing on PGHD integration in Electronic Health Records (EHRs); and considering policies for improving health information interoperability. This multi-faceted approach requires technical, ethical, legal, and policy considerations for successful PGHD integration while ensuring data privacy and security.

RQF1 distinctly highlights the security measure that could arise from the extensive use of AI and ML in healthcare. In this context, we have pinpointed a range of security measures, varying from basic [3,23,24,77,84,85,95,96,97,98,99,100,101] to advanced [62,102,103,104,105,106,107,108,109,110,111], which can be effectively implemented to ensure the secure usage and management of PGHD. RQF2 [6,21,23,24,25,26,27,37,53,58,73,77,78,79,81,85,95,112] suggests that the development of wearable devices and internet-connected medical devices over the next decade will profoundly impact PGHD security and privacy. Key influences include a massive increase in PGHD volume, advanced data analytics, improved security measures, new regulations, greater public awareness, systems integration challenges, ethical implications, market-driven solutions, personalized security settings, and global disparities. While projections are uncertain, it is likely the confluence of technological, regulatory, and societal changes will reshape how PGHD security and privacy concerns are addressed. Companies and policymakers should proactively develop robust, flexible frameworks to keep pace with these expected changes.

## 7. Conclusions

In conclusion, the evolving landscape of PGHD that ranges from social media data to physiological data presents both transformative opportunities and significant challenges in the realm of healthcare. The substantial increase in the variety and interconnectedness of the patient-generated data, which are seen as PGHD, improves the ability to predict individual behaviors, health conditions, and diseases. But in many countries, laws particularly safeguard health-related data, recognizing its sensitivity. The current extensive integration of diverse data sets, which can include a wide range of data landscapes, has the potential to reclassify data not traditionally viewed as health-related into sensitive health information [113]. The integration of PGHD into healthcare models also offers profound benefits, including enhanced patient engagement, improved accuracy of health records, and more personalized care. However, this integration is not without its complexities, particularly with regard to the security and privacy of sensitive health data. The key challenges identified include ensuring the authenticity and accuracy of PGHD, seamlessly integrating these data into existing healthcare systems, and addressing the substantial privacy and security concerns associated with digital health data. The article underscores the critical need for robust security measures, including end-to-end encryption, secure data transmission protocols, and stringent access controls, to safeguard against unauthorized access and cyber threats. Furthermore, the article highlights the importance of developing comprehensive privacy frameworks and governance models that prioritize patient consent and transparency. This includes the need for continuous adaptation to emerging technologies like AI and machine learning, which require novel approaches to data security and privacy. To optimize the value of PGHD while protecting patient privacy and data integrity, a collaborative effort is required among stakeholders, including healthcare providers, technology developers, policy makers and patients themselves. This involves not only technological solutions but also ethical, legal, and policy considerations. The dynamic nature of PGHD, coupled with the rapid advancement of technology, requires ongoing research and development in this field. Future directions should focus on developing innovative security techniques, exploring new models for effective data integration, and continuously updating regulatory frameworks to keep pace with technological advancements. In essence, the successful and secure integration of PGHD into healthcare models hinges on a multifaceted approach that combines technological innovation with strong policy frameworks, ethical considerations, and active stakeholder engagement. This approach will be crucial to realize the full potential of PGHD in enhancing healthcare delivery and patient outcomes while maintaining the highest standards of data privacy and security.

## Figures and Tables

**Table 1:** Difference between the Patient-Generated Health Data (PGHD) and Clinical data.

Factor	PGHD	Clinical Data
Source of Data	Generated by patients or caregivers using patients devices, apps, or self-reporting.	collected by healthcare professionals during visits, hospital stays, or by diagnostic procedures.
Accuracy and Reliability	Can vary; depends on the accuracy of the device and the patient’s ability to accurately record data.	Generally high, collected through validated methods and equipment.
Scope and Context	Provides a broader view of the patient’s health in daily life, including lifestyle and environmental factors.	Focuses on health status during clinical assessments; may miss daily life nuances.
Usage in Healthcare	Used for remote monitoring, chronic disease management, and patient participation in care.	Fundamental for diagnosis, treatment planning, and disease monitoring.
Integration Challenges	Integration with clinical workflows and EHRs can be challenging due to data variability.	Usually well-integrated, though interoperability between different EHR systems can be an issue.
Patient Engagement	Encourages active patient participation and self-management.	Typically, more passive, with healthcare professionals driving the process.

**Table 2:** Articles included on PGHD Understsnding, Requirement, Challenges Review.

Category	Subcategory and References
PGHD	Research Question Exploratory
	RQE1 [6,26,33,58,65,66,67]
	RQE2 [3,7,68,69,70,71]
	Research Question Impact Assessment
	RQI1 [72]
	RQI2 [6,35,73,74,75,76]
	Research Question Solution-Oriented
	RQS1 [76,77,78,79,80,81,82,83,84,85]
	RQS2 [3,6,7,86,87,88,89,90,91,92,93,94]

**Table 3:** Articles included on PGHD Basic and Advanced Security and Privacy Review.

Category	Subcategory and References
PGHD Security & Privacy	Research Question Future-Oriented
	RQF1: Basic Security Measure [3,23,24,77,84,85,95,96,97,98,99,100,101]
	RQF1: Advanced Security Measure [62,102,103,104,105,106,107,108,109,110,111]
	RQF2 [6,21,23,24,25,26,27,37,53,58,73,77,78,79,81,85,95,112]

**Table 4:** Primary Source and Utilization of PGHD in Healthcare Settings.

Article	Primary Sources	Purpose in Healthcare Settings
Bourke et al. [59]	Social media, Mobile apps, Online surveys, Speech recordings, Frequency of social communication	Gain person-centric insight not available in routine healthcare datasets, Enable better self-management and improve health outcomes, Support healthcare professionals in monitoring and managing patients, Improve relationships and communication with healthcare teams, Augment patient-driven quality of care assessment, Provide additional information to inform research in healthcare settings
Austin et al. [95]	Sensor devices, Mobile technologies	Enhance care delivery and outcomes, Support research and clinical practice, Improve health outcomes, Facilitate more “connected health” between patients and care teams
Nittas et al. [67]	Active data generation combined with Passive sensor-based trackers	Utilized for primary disease prevention and health promotion purposes, Used for exercise-based weight loss, well-being promotion, and healthy aging, Embedded in larger multicomponent preventive interventions, Combined with reflective, process guiding, motivational, and educational components
Lai et al. [66]	Mobile health technologies, Consumer-grade devices	Collecting and monitoring physiological signs of chronic conditions, Facilitating self-management of Inflammatory Bowel Disease (IBD), Monitoring patients in real-life settings through video surveillance, Integrating continuous glucose monitor data for Personal Health Record (PHR), Incorporating PGHD into clinical trials for more accurate patient information, Monitoring medication adherence and reminding patients to follow study protocols
Lavallee et al. [7]	Wearable devices, Mobile health apps, Geolocation technologies	To support care decisions and improve patient-provider communication and engagement, To expand care for individuals with limited access to healthcare, To improve care for those with acute or chronic conditions, To better engage patients in the use of patient portals and electronic health records, To ensure clinical data accurately reflects the health status of patients, To understand behaviors and health risks for better population health support
Petersen et al. [34]	Remote sensors, Smart wearable devices, Social media, Mobile health (mHealth) apps	Shift in medicine practice towards patient experience-based outcomes, Evaluation and choice of treatment options, Assessment of asthma control, Customized care plans and prediction of length of stay, Assessment of nutritional status
Kawu et al. [27]	mHealth apps, Wearables, Social media posts	Aid in decision-making and provision of personalized care, Provide additional information for healthcare professionals, Assist in the development of reimbursement structures, Facilitate the flow and use of PGHD by clinicians, Enable informed decisions about developing and sharing PGHD

**Table 5:** Patient Perceptions and Utilization of PGHD.

Article	Patient Perceptions	PGHD Utilization	Key Findings
Zhu et al. [68]	Positive: gain insights, participate in care, Negative: burdensome to share in visits	Self-management, Share with clinicians to inform treatment decisions	Patients motivated to collect PGHD for self-awareness, self-management, track effectiveness of treatments, Want to use PGHD to alter treatment plans and medications, Perceive sharing PGHD as burdensome due to time constraints, Want guidance from clinicians on relevant data to collect
Lavallee et al. [7]	Positive if providers find it useful/relevant, Negative if providers not receptive	Share targeted PGHD to inform treatment decisions, Discontinue sharing if provider not receptive	Selective in sharing PGHD based on perceived provider interest and relevance, Consider visit time constraints when deciding what PGHD to share, Less likely to continue sharing if providers don’t find it useful
Burns et al. [69]	Positive: engaging, empowering	Self-monitoring health conditions, Share with providers to improve communication	Collecting PGHD engages patients, enhances doctor-patient relationships, Allows patients to feel empowered, reassured, in control, Provides visual evidence to accompany descriptions of health issues
Adler-Milstei et al. [3]	Positive: enhances understanding, assist providers, Negative: privacy concerns, lack of communication on use	Achieve health goals, Diagnosis and care management	Aligns with patient preferences except 3rd party biometric data (privacy concerns), Motivated to achieve health goals, enhance understanding, assist providers, Concerns about manual effort required, lack of communication on value and use of data, Stress integrating PGHD into interactions with providers
Kim et al. [70]	Mixed: benefits but concerns on privacy, autonomy	Inform clinical care (but potential for manipulation)	Enhances transparency but also raises patient concerns about privacy, autonomy, Questions around reliability of data, need for education on collection and use, Mismatch between available technology and actual patient/provider needs
Smith et al. [71]	Positive: altruistic motivation, Expect privacy, reciprocity	Share with cancer registries to aid research	Motivated to share to aid other patients (altruistic), Want confidentiality, reciprocal information on treatments, side effects etc., Preferred methods: mail, phone, online surveys, patient portals

**Table 6:** Impact on incorporation of patients and their PGHD in healthcare decision-making.

Article	Patient Incorporation	PGHD Incorporation
Jim et al. [73]	Allow care to be tailored to patient’s unique needs and preferences; Inform treatment choices; Predicts health events; Population health management; Clinical trial outcomes; Regulatory decisions; Remote patient monitoring	Improves symptom monitoring and management; Provides additional and more up-to-date information about the patient; Allows continuous monitoring of health parameters like medication adherence, exercise, sleep, etc.; Integrates PGHD and HPGD for a more holistic view of the patient’s health; Increases patient engagement and empowerment; Potentially improves quality of decision-making; Addresses challenges like data integration, privacy/security, regulatory issues
Singh et al. [74]	Facilitates patient-centered care; Enhances shared decision-making	Provides additional and more up-to-date information about the patient; Allows continuous monitoring of health parameters such as medication adherence, exercise, sleep, etc.; Integrates PGHD and HPGD for a more holistic view of the patient’s health; Increases patient engagement and empowerment; Potentially improves quality of decision-making; Addresses challenges such as data integration, privacy/security, regulatory issues
Petersen et al. [35]	Actively involved in collecting and sharing health data; Facilitates productive, informed consultations with healthcare providers; Contributes to greater engagement from patients; Encourages honesty in patient-provider interactions	Provides valuable insights between visits and reveals patient issues that may be missed during appointments; Provides comprehensive view of patient’s health over time; Empowers patients by giving them control over their health data; Enhances patient autonomy and transforms patient-clinician dynamic; Necessitates development of specific protocols for data collection and integration into EHR; Addresses privacy and security concerns
Wood et al. [75]	-	Enhances treatment efficacy; Identifies patients at risk of treatment toxicity; Informs personalized treatment plans; Contributes to evaluation of novel therapeutics; Tracks metrics to improve patient well-being
Petersen et al. [76]	Brings in the patient perspective; Empowers patients to take an active role in managing their health	Provides a more holistic view of patients for tailored care plans; Enables tracking of health status over time; Reveals health trends at the population level; Addresses issues like privacy risks, ensuring accuracy, and building clinician trust in PGHD; Suggests policy changes for transparent and ethical collection and use of PGHD

**Table 7:** Basic Security Measures for PGHD.

Security Measure	Detailed Description
Data Collection Policies [95]	Establish comprehensive policies that govern the collection of PGHD, ensuring that all data types, collection methods, and data flows are well-defined and documented. These policies should also dictate the lifecycle of the data from collection to deletion, including use cases for AI and ML applications.
Consent and Transparency [23]	Implement mechanisms to obtain explicit patient consent for the use of their PGHD in AI and ML applications, providing transparency regarding the purpose of data collection, processing, and sharing. This involves clear communication with patients about their data rights and the measures in place to protect their privacy.
Secure Data Transmission [96]	Utilize end-to-end encryption methods to secure the transmission of PGHD from patients’ devices to healthcare systems. This ensures that data remains confidential and is protected against interception or unauthorized access during transmission.
Robust Authentication [77]	Apply strong authentication measures to verify the identity of patients and healthcare providers accessing PGHD. This might include the use of multi-factor authentication (MFA), biometric verification, or unique patient identifiers.
Data Minimization [3]	Collect only the PGHD that is necessary for specific AI and ML healthcare applications. This approach minimizes the volume of sensitive data that could be compromised in a breach and limits exposure to only essential elements.
Data Storage Security [97]	Secure the storage of PGHD with encryption and other protective measures. Ensure that databases and storage environments adhere to health industry security standards and best practices, such as using encrypted databases and secure cloud services with health data compliance certifications.
Endpoint Security [98]	Strengthen the security of patient-owned devices that generate PGHD, including mobile phones, wearables, and home monitoring devices. This may involve the deployment of security software, regular security updates, and secure configuration guides for patients.
Network Security [99]	Deploy comprehensive network security solutions, including firewalls, intrusion detection/prevention systems, and secure Wi-Fi protocols, to protect the networks over which PGHD is transmitted. Regularly monitor network traffic for anomalies and conduct penetration tests to detect vulnerabilities.
Data Anonymization [100]	When using PGHD for AI and ML training, apply data anonymization techniques to remove or obfuscate personal identifiers, reducing the risk of patient re-identification. This can involve techniques such as differential privacy, where “noise” is added to the data to prevent individual identification while still maintaining the data’s utility for analysis and model training.
Regular Security Audits [84]	Perform periodic security audits and risk assessments on systems handling PGHD to ensure compliance with policies and to identify any new threats or vulnerabilities. This can help in proactively addressing security gaps and updating defenses accordingly.
Data Integrity Checks [24]	Implement checksums, digital signatures, and other data validation methods to ensure the integrity of PGHD throughout its lifecycle. This helps in detecting any unauthorized alterations or corruption of data, which is crucial for the accuracy and reliability of AI and ML outputs.
Secure Application Development [101]	Adhere to secure software development life cycle (SDLC) practices when developing AI and ML applications that process PGHD. This includes regular code reviews, security testing, and the incorporation of security by design principles to minimize vulnerabilities in applications that will handle sensitive health data.
Disaster Recovery and Business Continuity [84]	Develop and maintain comprehensive disaster recovery and business continuity plans to ensure the resilience of systems managing PGHD. These plans should provide for the rapid restoration of data and services in the event of an incident, with minimal disruption to healthcare services and patient care.
Legal and Regulatory Compliance [23]	Stay informed and compliant with all relevant legislation and regulations concerning PGHD, such as HIPAA, HITECH, GDPR, and other regional data protection and privacy laws. Compliance should be continuously monitored and updated in response to legislative changes, especially those related to the use of PGHD in AI and ML applications.
Training and Awareness [85]	Regularly conduct comprehensive training programs for all stakeholders involved in the collection and processing of PGHD, including healthcare professionals, IT staff, and patients. This training should focus on the importance of data security, privacy best practices, and the proper use of AI and ML applications in healthcare.

**Table 8:** Advanced Security Measures for PGHD.

Security Measure	Detailed Description
Federated Learning [102]	Implement federated learning to train AI models on decentralized data. In this approach, the data remains on the patient’s device or local server, and only model updates are shared to a central server for aggregation. This reduces the amount of PGHD transferred and stored centrally, thus minimizing exposure and enhancing privacy.
Blockchain Technology [62]	Utilize blockchain technology to create a secure, immutable ledger of transactions related to PGHD. This can be used to manage consent, track data access, and ensure data integrity. Since blockchain records are tamper-evident and decentralized, they provide a robust framework to audit and verify the provenance and handling of PGHD, ensuring transparency and trust in the system.
Homomorphic Encryption [103,104]	Apply homomorphic encryption to allow computation on encrypted PGHD, enabling AI and ML analysis without decrypting sensitive data. This type of encryption allows data to remain encrypted even during the analysis process, greatly reducing the risk of data exposure. AI models can be trained and refined using encrypted data, thus preserving patient privacy throughout the AI lifecycle.
Secure Multi-party Computation [105]	Engage in secure multi-party computation (SMPC) protocols to enable a group of parties to jointly compute a function over their inputs while keeping those inputs private. This could allow for collaborative AI model training or data analysis on PGHD without exposing individual patient data to any of the involved parties, including the model trainer.
Differential Privacy [106]	Incorporate differential privacy techniques in the data analysis and AI training processes to add statistical noise to PGHD. This approach ensures that the output of the analysis or the behavior of the trained model does not reveal sensitive information about any individual, thereby providing a quantifiable level of privacy.
Zero-Knowledge Proofs [107]	Integrate zero-knowledge proof systems to enable the verification of data integrity and authenticity without revealing the underlying data. This can be particularly useful in verifying patient identities and the authenticity of PGHD without exposing the actual data, ensuring that only necessary data is processed and reducing the risk of privacy breaches.
Advanced Persistent Threat (APT) Protection [108]	Adopt APT protection strategies to defend against sophisticated, prolonged cyber-attacks aimed at stealing data. This involves a combination of advanced security technologies and practices such as network segmentation, behavioral analytics, threat intelligence, and proactive hunting for threats to protect PGHD and the systems that process it.
AI Security Audits [109]	Conduct specialized security audits focusing on the AI components involved in processing PGHD, examining the model’s robustness against adversarial attacks, data poisoning, and other AI-specific threats. This also includes ensuring the AI system’s transparency and explainability, which is vital for trust and compliance in healthcare applications.
Decentralized Identity Management [110]	Deploy decentralized identity management systems that give patients control over their digital identities and the sharing of their PGHD. Such systems can employ blockchain technology and support selective disclosure, allowing patients to prove certain aspects of their identity or health data without revealing more information than necessary.
Continuous Security Monitoring [111]	Establish continuous security monitoring practices, using advanced security information and event management (SIEM) systems, to detect and respond to anomalies in real time. This is crucial to protect the integrity of PGHD during collection, storage, and processing for AI and ML applications.

## Data Availability

Data not available. The current study did not generate any new data.
